# Dynamics of localised nitrogen supply and relevance for root growth of *Vicia faba* (‘Fuego’) and *Hordeum vulgare* (‘Marthe’) in soil

**DOI:** 10.1038/s41598-020-72140-1

**Published:** 2020-09-25

**Authors:** Sebastian R. G. A. Blaser, Nicolai Koebernick, Oliver Spott, Enrico Thiel, Doris Vetterlein

**Affiliations:** 1 Department of Soil System Science, Helmholtz-Centre for Environmental Research GmbH – UFZ, Theodor-Lieser-Str. 4, 06120 Halle (Saale), Germany; 2 Institute of Agricultural and Nutritional Sciences, Martin-Luther-University Halle-Wittenberg, Von-Seckendorff-Platz 3, 06120 Halle (Saale), Germany; 3 Agricultural Application Research, SKW Piesteritz GmbH, Am Wieseneck 7, 04451 Cunnersdorf, Germany

**Keywords:** Tropism, Element cycles, Agroecology

## Abstract

Root growth responds to local differences in N-form and concentration. This is known for artificial systems and assumed to be valid in soil. The purpose of this study is to challenge this assumption for soil mesocosms locally supplied with urea with and without nitrification inhibitor. Soil column experiments with *Vicia faba* (‘Fuego’) and *Hordeum vulgare* (‘Marthe’) were performed to investigate soil solution chemistry and root growth response of these two species with contrasting root architectures to the different N-supply simultaneously. Root growth was analysed over time and separately for the fertiliser layer and the areas above and below with X-ray CT (via region growing) and WinRHIZO. Additionally, NO_3_^−^ and NH_4_^+^ in soil and soil solution were analysed. In *Vicia faba*, no pronounced differences were observed, although CT analysis indicated different root soil exploration for high NH_4_^+^. In *Hordeum vulgare*, high NO_3_^−^ inhibited lateral root growth while high NH_4_^+^ stimulated the formation of first order laterals. The growth response to locally distributed N-forms in soil is species specific and less pronounced than in artificial systems. The combination of soil solution studies and non-invasive imaging of root growth can substantially improve the mechanistic understanding of root responses to different N-forms in soil.

## Introduction

Root system architecture (RSA) is an important property that can be influenced by the availability of nutrients and their concentration and distribution in the growth medium^1–5^. This is a well-known phenomenon for phosphorus^6–8^, but also of high interest for nitrogen (N), not least due to the agricultural application of fertilisers with different N-species composition. One general conclusion from studies addressing root growth response to different distributions or ratios of NO_3_^−^ and NH_4_^+^ was that NH_4_^+^ nutrition results in a higher number of lateral roots, while NO_3_^−^ nutrition results in increased elongation of laterals. Nacry et al.^9^ specified additionally, two very general aspects that hold true for most circumstances: (1) high N status of the plant leads to a systemic repression of lateral root growth and (2) exogenous NO_3_^−^ or NH_4_^+^ stimulates lateral root growth locally. However, a typical root growth response to N that is applicable for all species and conditions is almost impossible to define, as many biotic and abiotic factors are responsible for the final shape of the RSA^9^.

Many of the existing studies about root growth response to local availability of nutrients used approaches with nutrient solution^10–20^ or agar plates^21–25^. These studies pointed out that RSA can be modulated in an intense manner, given the right experimental conditions. The well-known studies from Drew and colleagues^7,14,26–28^ likewise showed a marked response of barley roots to NO_3_^−^ and NH_4_^+^ in column experiments. In Drew^26^ quartz sand was used as growth medium and wax membranes were installed to separate the columns in three layers percolated with nutrient solution differing in concentration of the respective nutrient.

It is uncontroversial that experimental conditions in laboratory studies cannot exactly represent natural conditions inasmuch as they usually serve as simplified model systems. When studying RSA in nutrient solution, agar plates, or even artificial soils (e.g. quartz sand) some significant aspects of plant-soil interaction remain largely unconsidered. The results from these studies using artificial systems vary considerably and one has to keep in mind that in these simplified systems applied concentrations are typically low and constant, as is the pH due to added buffers or frequent exchange of the nutrient solution. Furthermore, sorption is not a factor there and the root surface is usually in direct contact with the nutrient solution, which is quite different from conditions found in soils. Moreover, organic matter is always an additional source of N supply and also microbial activity in soils is highly diverse and dynamic resulting in a high variability of turnover rates for hydrolysis of urea and for nitrification^29,30^. Furthermore, NO_3_^−^ and NH_4_^+^ normally co-exist in soils at the same time but concentration and mobility may differ by orders of magnitude^31^. In addition, NO_3_^−^ is highly mobile but NH_4_^+^ is largely adsorbed to the soil matrix, with sorption depending strongly on many factors, e.g. clay mineral composition, clay content, soil organic matter content and the amount of competing cations^32,33^. Growing roots in other growth media than soil decreases experimental variability as several biotic and abiotic factors that can influence the shape of root morphology like mechanical impedance, drought or plant growth promoting bacteria are neglected^34^.

There are studies that have been performed in soil^11,15,16,35–46^, which found widely differing results of plant growth response to different N applications, covering the whole spectrum from no response to stimulation of root growth to inhibition and toxicity symptoms. However, measurement of N-dynamics in soil and soil solution (i.e. change of concentration, distribution and N-form) was largely neglected, as was root growth analysis over time in most of these studies. Moreover, most studies only used one plant species, although it is known that root growth responses are not uniform across species^2,46^.

The present study therefore aims to overcome some of the shortcomings of previous experiments by analysing RSA response to N supply in a more realistic soil environment. The response of the root system to heterogeneous availability of nutrients is reflected in both physiological and root growth plasticity^2^. However, this study only refers to root growth plasticity. The goal is to enhance the understanding of temporal and spatial dynamics of root response in situ, in the soil to different N forms. For this purpose we used two different plant species with contrasting RSA, a real soil substrate (i.e. including sorption capacity, soil organic matter and microbial N turnover) as growth medium and commercial urea fertiliser granules with and without a nitrification inhibitor (NI) in order to create treatments varying in NO_3_^−^ and NH_4_^+^ concentrations. Urea was used as it is the most widely applied nitrogen fertiliser on a global scale according to the Food and Agriculture Organization of the United Nations (FAO). In soil, urea is rapidly hydrolysed to NH_4_^+^ which is subsequently adsorbed to clay particles and organic matter or further oxidised to NO_3_^−^ if the relevant microorganisms are present and active. This oxidation process is delayed by the use of NI^47–49^. As a consequence, NH_4_^+^ availability in the soil and thus, its importance as a nutrient is increased. The well-known issue that roots growing in soil cannot be observed directly is overcome by application of X-ray computed tomography (CT), at least for *Vicia faba*. X-ray CT is currently one of the best non-invasive imaging techniques for visualising and quantifying plant roots in soil *in-situ* over time^50^.

With this work, we show that responses to naturally occurring heterogeneities in N supply after fertilisation may also affect the root architecture of major crops albeit to a different extent compared to studies with a very steep gradient in nutrient concentration and without a genuinely nutrient-deficient zone the roots have to grow through first before reaching the patch with higher nutrient content. The literature is controversial about the reason for the morphological root reactions to N, as it is generally a transient nutrient and morphological reactions are often slow and energetically expensive^2,9,13,16,25,26,38,46^.

## Material and methods

### Experiment 1: test of inhibitor functionality and fertiliser turnover under standardised laboratory conditions

For all experiments in this study a silty clay loam soil, originating from the subsoil of a haplic Luvisol (40–65 cm depth) was used. This was done, as the background concentrations of NO_3_^−^ and NH_4_^+^ in soil solution were rather low in this subsoil, i.e. 2–4 mM NO_3_^–^-N and 0.2–0.3 mM NH_4_^+^-N, respectively^51^. Total N content of the soil without addition of fertilisers was 690 mg kg^−1^^51^. CEC of the soil was 170.6 (± 9.1) mM_c_ kg^−1^ and C_org_ was low with 0.5 (± 0.01)%^52^. For more details of the soil, see Vetterlein et al.^52^.

From this sieved (< 2 mm) and homogenised soil material, 300 g were mixed with 30 mg N in form of ground urea granules (treatment “U”, product name PIAGRAN^®^ 46, SKW Piesteritz) or ground urea granules containing the nitrification inhibitors 1H-1,2,4-triazol (0.09%) and DCD (0.91%) (treatment “U+NI”, product name ALZON^®^ 46, SWK Piesteritz), both of which are commercial and approved EU-fertilisers. Water was added to obtain 50% of maximum water holding capacity (180 ml kg soil^−1^, equal to 18% w/w). This mixture was split into three replicates at 100 g of soil each, filled in bottles and analysed separately. Closed bottles (loosely closed by plastic foil and rubber band) were kept at constant temperature of 20 °C over a period of 67 days. Samples were taken at days 0, 1, 2, 3, 7, 10, 14, 21, 28, 35, 43, 49, 56 and 67, subsequently extracted with 1 M KCl by headlong shaking for 1 h^53^. Concentration of NH_4_^+^ was measured photometrically (AA3, Seal Analytical) to test the functionality of the nitrification inhibitor and to monitor urea turnover under controlled laboratory conditions without plant influence in order to give a rough estimation of the temporal dynamics for the subsequent plant experiments.

### Experiment 2: fertiliser turnover under controlled climate chamber conditions, measured in soil solution over time and soil extraction after 38 days

Acrylic columns (250 mm height, 35 mm radius, 5 mm wall thickness) were filled with 1,050 g of sieved (< 2 mm) and homogenised silty clay loam described before and packed to a bulk density of 1.2 g cm^−3^. The uppermost 2–3 cm of the columns were left unfilled to enable addition of coarse gravel. The coarse gravel served as a capillary barrier to prevent capillary water movement to the soil surface and by this to prevent excessive evaporation from the soil surface.

Three treatments were set up: (1) control = “C” without any fertiliser input; (2) urea without any additives = “U”; and (3) urea with nitrification inhibitor = “U+NI”. Both N-fertilised treatments received five fertiliser granules of similar size per sample. Urea granules are very easily soluble: in water they dissolve within minutes and in moist soil within a few hours at most. Urea is known to be hygroscopic and hence, forced to dissolve even by limited water availability. Due to these properties, a similar dissolution behaviour as with the ground granules in experiment 1 can be expected. The rate was 100 mg N kg^−1^ soil, placed in one layer 5–6 cm below soil surface and arranged equidistantly. These 100 mg N per kg account for about 13% of total N related to the background N of 690 mg kg^−1^ soil. Two replicates per treatment were set up, as no considerable additional variation was expected due to plant absence.

The soil was initially watered with 177 ml distilled water, resulting in a water content of about 27 vol.-% (= pF 2.65, equal to − 0.45 kPa). First, 137 ml were supplied from below by capillary rise and, as soon as the water level reached the fertiliser layer, 40 ml were carefully supplied from above to prevent excessive transport of the very mobile urea out of the fertiliser layer towards the soil surface. Coarse gravel was placed on top of the soil to reduce evaporation. Each column was placed on a weighing cell to keep water content in each column as stable as possible by rewatering every second day. Soil columns were wrapped with aluminium foil to prevent algae growth. Soil columns were set up in a climate chamber in a randomised block design under controlled conditions (12/12 h day/night cycles at 19 and 16 °C, respectively; photosynthetic active radiation was 350 µmol m^−2^ s^−1^, relative air humidity was 65%). Samples were incubated for 3 weeks before the micro suction cups were installed.

Soil chemical conditions in soil solution were measured over time with micro suction cups (‘MicroRhizons’, Rhizosphere Research Products B.V., The Netherlands), installed in all six columns in two different soil depths. Soil solution was only extracted from the experiment with *Vicia faba*, due to technical problems during the experiment with *Horduem vulgare*. The upper layer was located in the plane of fertiliser placement. The second layer was installed 5 cm below, as a control for monitoring potential N transport. In each layer, three suction cups were installed. All suction cups were connected to thin Teflon tubes, attached to a chamber equipped with 2 ml vessels for each tube. These chambers were set to negative pressure (− 40 kPa) for about one hour per sampling date to collect soil solution. Sampling was conducted on day 20, 28, 32 and 36 after start of incubation. In relation to the plant growth experiment these dates correspond to − 1, 7, 11 and 15 DAP (days after planting—this is the reference time scale used in all figures). Soil solution samples from individual suction cups in the same layer were combined to one mixed sample representative for the respective soil layer. Soil solution was analysed for pH (IQ240, I.Q. Scientific instruments, Inc., USA), osmotic potential (Osmomat 030, Gonotec, Germany), NO_3_^–^-N and NH_4_^+^-N (measured photometrically, AA3, Seal Analytical).

After 36 days, soil columns were cut with a ceramic knife in horizontal layers of 3 cm thickness, resulting in 7 layers per sample. These samples were analysed for mineral N (NO_3_^−^ and NH_4_^+^). The mineral N fraction extracted by 1 M KCl^53^ comprises N in solution and extractable N at the exchange sites of the soil like clay minerals and organic matter. Strongly bound or fixed NH_4_^+^ in the interlayers of clay minerals are not completely extracted with this method. This fraction is henceforth referred to as the sum of readily available and adsorbed N species, while N species in soil solution are referred to as readily available fractions.

### Experiment 3: plant growth and root response of *Vicia faba* (‘Fuego’) and *Hordeum vulgare* (‘Marthe’) to different soil chemical conditions and N-forms in soil, resulting from urea fertiliser with and without nitrification inhibitor, over time

Experiment 3 was set up as randomised block design with the same treatments and conditions as experiment 2 regarding soil preparation, fertiliser application, climate chamber conditions and extraction of soil solution. The major difference was the presence of plants.

### Plant material

Faba bean (*Vicia faba* L., cv. ‘Fuego’) and barley (*Hordeum vulgare*, cv. ‘Marthe’) were used in this experiment. The rationale for this was to test two arable crop species with contrasting root system architectures for their growth response to the same experimental conditions. It is known that root growth responses are not uniform across species^2,46^. *Vicia faba* was chosen as with its large root diameters the challenge of following the development of individual laterals could be addressed^54^. In this way, development of RSA for different root orders was captured^55,56^. However, as *Vicia faba* is not relevant for N fertilisation purposes in agricultural practice, we added *Hordeum vulgare* as a second species.

Not inoculated seeds of faba bean and barley were surface sterilised with H_2_O_2_ (10%) for 10 min and soaked in saturated CaSO_4_ for about four hours before one seed was placed per column about 2 cm below soil surface. Seed planting was performed at day 21 after start of soil incubation (= 0 DAP). Plants were grown for 17 days.

Four replications were set up for *Vicia faba*, 12 replications for *Hordeum vulgare*. The reason for different numbers of replicates was the chosen method to acquire data about root growth dynamics over time. For *Vicia faba*, X-ray computed tomography (CT) was used^55^. For *Hordeum vulgare*, having much finer root diameters, X-ray CT at the given column diameter would have captured only seminal roots and first adventitious roots in the soil utilised in the present experiment. The soil was characterised by small aggregates and hence high heterogeneity in grey values caused by the mix of small intra-aggregate pores and larger inter-aggregate pores would have hindered segmentation of roots from X-ray CT images. Hence, in the same frequency as X-ray CT was performed for *Vicia faba*, four replicates of *Hordeum vulgare* treatments were harvested destructively. Roots were washed out and analysed with WinRHIZO 2009b (Regent Instruments Inc., Canada). Destructive harvest was also conducted for *Vicia faba* at the end of the experiment (17 DAP).

### CT scanning configuration and image analysis

X-ray tomography was performed with an industrial µCT (X-TEK XTH 225, Nikon Metrology) with 140 kV, 286 µA (equal to 40 Watts) and 500 ms exposure time. Each scan was performed with 1,000 projections and one frame, resulting in an exposure time of 8.5 min per scan. A copper filter with 0.5 mm thickness was used to reduce the beam hardening artefact. Distance between X-ray source and sample was 17.7 cm. The spatial resolution of the X-ray tomogram was 40 µm in the field of view. The calculated dose rate for these settings was 259 R h^−1^ (calculated with the Rad Pro Calculator for Desktop PCs Version 3.26 from https://www.radprocalculator.com/RadProDownloads.aspx). This equals 227 rad h^−1^, or 2.3 Gy h^−1^. Two tomograms one above the other were performed per sample in order to visualise and analyse the uppermost ~ 14 cm of the root systems in the soil in its true spatial arrangement. X-ray CT was performed on 8, 12 and 16 DAP. The cumulative scanning times and X-ray doses per sample are 51 min and 2.0 Gy, respectively. This dose is lower than in the low radiation treatment in Blaser et al.^55^.

A detailed analysis of CT-images was conducted for *Vicia faba*. Main points are stated here, for more details see supporting information in Blaser et al.^55^. Raw data was filtered (Gauss, kernel size 5) to reduce image noise. Root systems were segmented with semi-automated region growing in VG Studio Max 2.1. Based on the idea of Flavel et al.^57^, binarised root systems were skeletonised and analysed with the plugin BoneJ^58^ in the software Fiji^59^. The resulting information was used to distinguish between tap root, first and second order lateral roots.

A distance map was performed for the X-ray data, according to Schlüter et al.^56^. Main steps are given here, for more detailed information see Schlüter et al.^56^. 3D Euclidian Distance Transform was performed in Fiji, as suggested in Koebernick et al.^54^. For each slice, the histogram for the soil-root-distance was retrieved. The cumulated histograms depict the relative frequency of distances from the soil towards the nearest root voxel. With this parameter we can describe the exploration of the given soil volume by the root system. The distance map was performed for three separate layers within the X-ray CT stack, referring to the layers of destructive harvest for *Hordeum vulgare* (see below).

### Shoot and root analysis

Shoot weight and leaf area were measured directly after harvest. Leaf area was measured with WinRHIZO. Roots were washed out carefully with a sieve (2 mm mesh size) for several minutes per sample. Roots were stored in Rotisol and analysed with WinRHIZO a few days later. For *Hordeum vulgare*, soil columns were cut in three parts during each harvest. The first layer represented the part above the fertiliser plane and was 0–4 cm below soil surface. The second layer represented the fertiliser plane and was 4–9 cm below soil surface. The third layer represented the part below the fertiliser plane and consisted of the remaining 9–23 cm in depth. Roots were washed out carefully and analysed separately for each layer with WinRHIZO.

WinRHIZO analysis was performed for total root length and three functional diameter classes. For *Vicia faba*, thresholds were set at 0.75 and 1.25 mm with the intention to separate first and second order laterals from tap roots, based on root diameters. The smallest class below 0.1 mm was discarded, as it was error-prone due to root hairs causing misclassification.

For *Hordeum vulgare,* thresholds for functional diameter classes (seminal roots, first and second order lateral roots) were selected individually for each sample. This was necessary, as the root diameters changed during growth and therefore two fixed values for all points in time and soil depths would have led to low quality of analysis. Thresholds between seminal roots and laterals ranged between 0.175 and 0.425 mm. Thresholds between first order laterals and second order laterals ranged between 0.080 and 0.180 mm. Segments with diameters below 0.04 mm were discarded, as they were distorted due to root hairs. Additionally, the number of lateral roots was counted manually by eye for all layers and points in time, apart from the C-layer at 16 DAP due to the very high number of roots in those samples.

### Statistics

For all experiments, statistics were performed with SPSS 22 (IBM). The urea turnover experiment in experiment 1 and the influence of the plants on the pH at the end of experiment 3 were analysed with the T-test for independent samples taking into account the Levene's test.

The temporal dynamics of the pH values and N-forms in the soil solution were analysed with a one-way ANOVA with repeated measurements with regard to the four sampling dates and Bonferroni adjustment of the confidence interval taking into account the Mauchly’s test for sphericity and if necessary the Greenhouse–Geisser correction method. All remaining analyses were evaluated with one-way ANOVA and post-hoc Bonferroni tests to compare the treatments. This was always done separately for all time points and soil depths. All ANOVA performed were one-way without interaction term.

For the pH values, the statistical tests were carried out using the proton concentration. Standard errors are given in all figures as error bars. Significant differences (*p* < 0.05, unless stated otherwise) are indicated by different letters or asterisks (*) in the figures. No significant difference is indicated by “ns”.

## Results

### Experiment 1: characterisation of urea hydrolysis and nitrification activity of the soil material under standardised conditions without plants

**Figure 1:**
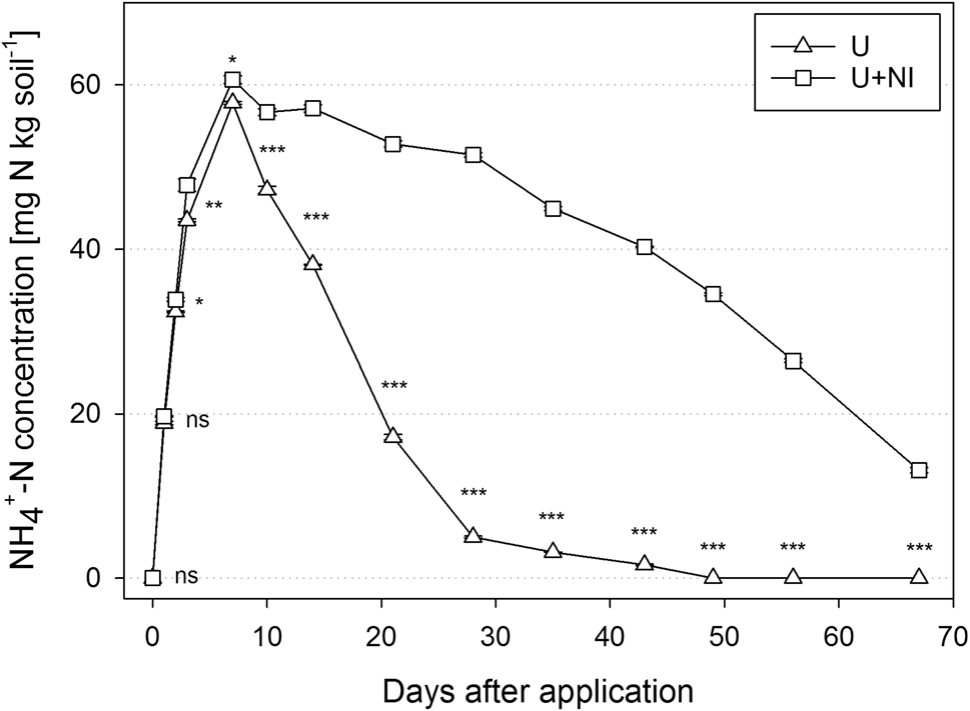
Temporal change of NH_4_^+^-N concentration under standardised conditions for treatments urea (“U”, triangle) and urea with nitrification inhibitor (“U+NI”, square) in soil extract (1 M KCl). Asterisk indicates significant difference between treatments at *p* < 0.05 (*), *p* < 0.01 (**) and *p* < 0.001 (***), ns = not significant. Error bars are masked by treatment symbols in most cases.

Experiment 1 revealed NH_4_^+^ formation by hydrolysis of urea and the subsequent depletion of NH_4_^+^ by nitrification. Both treatments were characterised by the same hydrolysis velocity, as the applied inhibitor selectively influenced nitrification. NH_4_^+^ concentration reached the highest level at day 7 for both treatments with a concentration of about 60 mg NH_4_^+^-N kg^−1^ soil (Fig. 1).

Urea hydrolysis is followed by nitrification provided that the relevant bacteria are present. The effect of the nitrification inhibitor was clearly shown, as NH_4_^+^ concentration decreased at a much slower pace in U+NI, compared to the pure urea treatment (U). The greatest differences regarding NH_4_^+^ concentration between both treatments were observed between days 20 to 30. Therefore, the incubation period for the later plant experiments was set to 21 days for a good trade-off between maximum difference in N-speciation, and high level of NH_4_^+^ in one of the treatments. The corresponding values for NO_3_^−^ are given in the supplementary material (Supplementary Fig. S1).

### Experiment 2: fertiliser turnover under controlled climate chamber conditions, measured in soil solution over time and soil extraction after 38 days without plants

#### Soil solution

**Figure 2:**
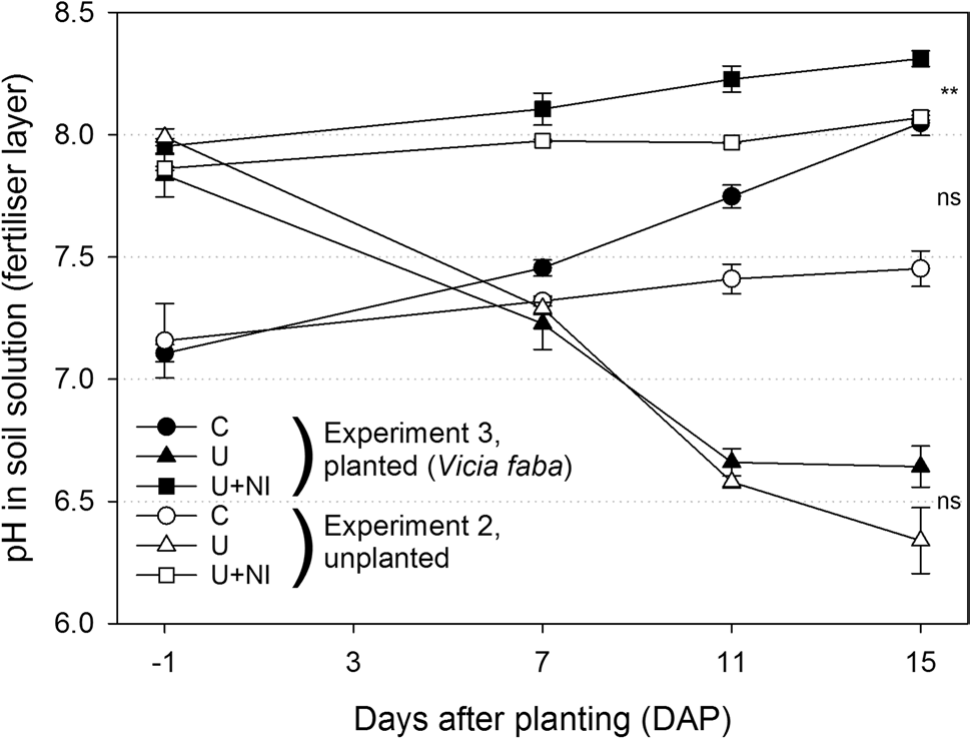
Change of soil solution pH with time in the fertiliser layer in experiments 2 (open symbols) and 3 (filled symbols) for treatments control (“C”, circles), urea (“U”, triangles) and urea with nitrification inhibitor (“U+NI”, squares). First sampling point (-1 DAP) defines starting conditions for root growth in experiment 3. Asterisk indicates significant difference between planted and unplanted treatments at *p* < 0.01 (**), ns = not significant. Error bars indicate standard error.

Dynamics of pH values (Fig. 2) reflected the turnover of applied urea-based fertilisers in experiment 2. In the control treatment (C), initial pH was close to 7 and did not change significantly over time (*p* > 0.05). In both fertilised treatments (U and U+NI) initial pH was higher (around 8) due to preceding urea hydrolysis. Thereafter, both treatments showed completely different developments. In U, pH steadily decreased over time to values around 6.5 at 12 DAP (*p* < 0.05). In U+NI, pH was stable around pH 8 and did not change over time (*p* > 0.05).

The pH-dynamics were similar to those found in the fertiliser layer in the treatments C and U+NI in the layer 5 cm below the fertiliser. A decreasing pH in the treatment U was not observed 5 cm below the fertiliser placement (Supplementary Fig. S2).

**Figure 3:**
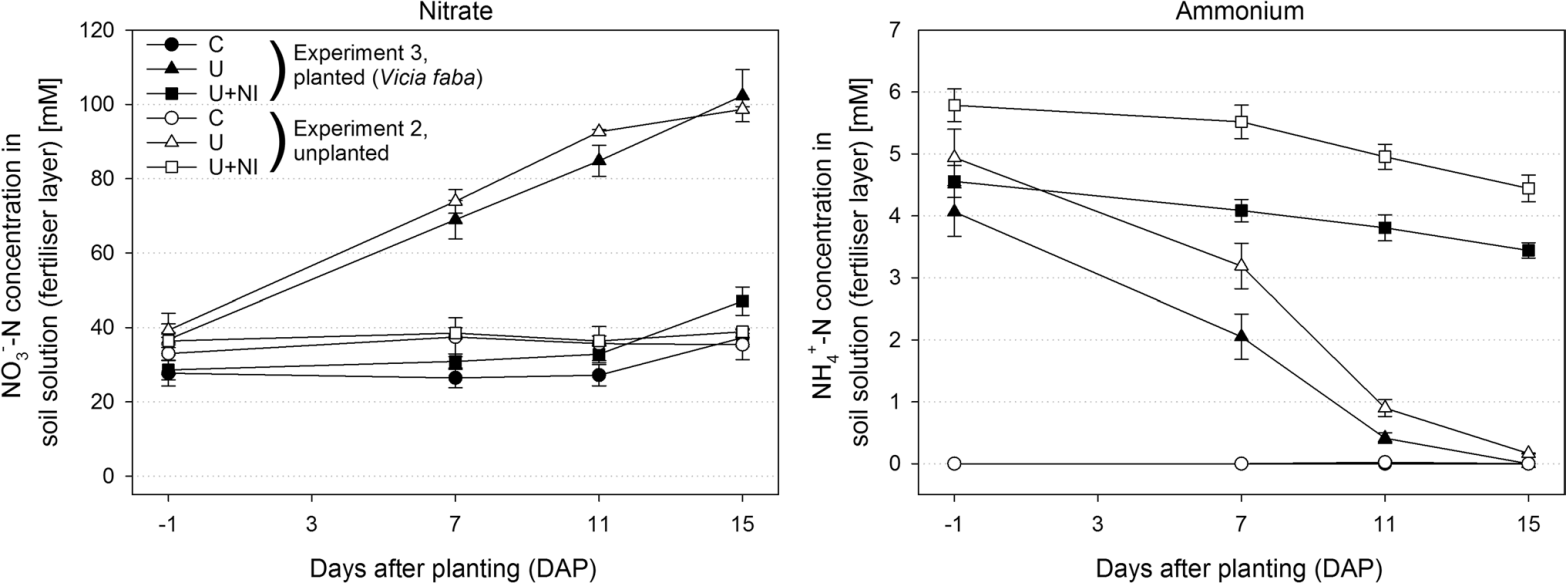
Temporal change of NO_3_^–^-N (left) and NH_4_^+^-N (right) concentration in soil solution in the fertiliser layer from experiments 2 and 3 after 3 weeks of incubation for treatments control (“C”, circles), urea (“U”, triangles) and urea with nitrification inhibitor (“U+NI”, squares). Filled symbols indicate plant presence (experiment 3), open symbols indicate plant absence (experiment 2). Starting point is one day before plants were introduced in experiment 3. Error bars indicate standard error.

Independent if planted or not, nitrification was the most striking transformation process in U, resulting in release of protons and decrease of pH. This is also reflected in the NO_3_^−^ concentrations in soil solution (Fig. 3). C and U+NI showed very similar and stable NO_3_^−^ concentrations around 30 mM. In contrast, NO_3_^−^ concentration in U increased throughout the duration of the experiment, reaching a maximum concentration of about 100 mM in the fertiliser layer by the end of the experiment. Linear correlations were found in U with (r^2^ = 0.99) and without plant (r^2^ = 0.98).

The NH_4_^+^ concentrations in soil solution confirmed the ongoing nitrification process in U. NH_4_^+^ concentration in the fertiliser layer decreased over time from about 4.5 mM to almost zero at the end of the experiment (Fig. 3). In U+NI, NH_4_^+^ concentration was stable around 4.5 mM during the first 7 days, reflecting the successful NH_4_^+^-stabilisation by the nitrification inhibitor. Subsequently, NH_4_^+^ concentration decreased slightly. In C, NH_4_^+^ was barely detectable. Linear correlations were found in both fertilised treatments, independent if planted (r^2^ = 0.98 in U and r^2^ = 0.99 in U+NI) or not (r^2^ = 0.96 in U and r^2^ = 0.90 in U+NI).

NH_4_^+^ concentration in soil solution below the fertiliser layer was below 1 mM. NO_3_^−^ concentrations were the same in C and U+NI as in the fertiliser layer, and much lower in the U treatment (Supplementary Fig. S3). The results of the osmotic potential were very similar to the results of NO_3_^−^, showing the same slope in U and a constant pattern in the treatments C and U+NI (Supplementary Fig. S4).

#### Absorbed and readily available N species in soil extraction

**Figure 4:**
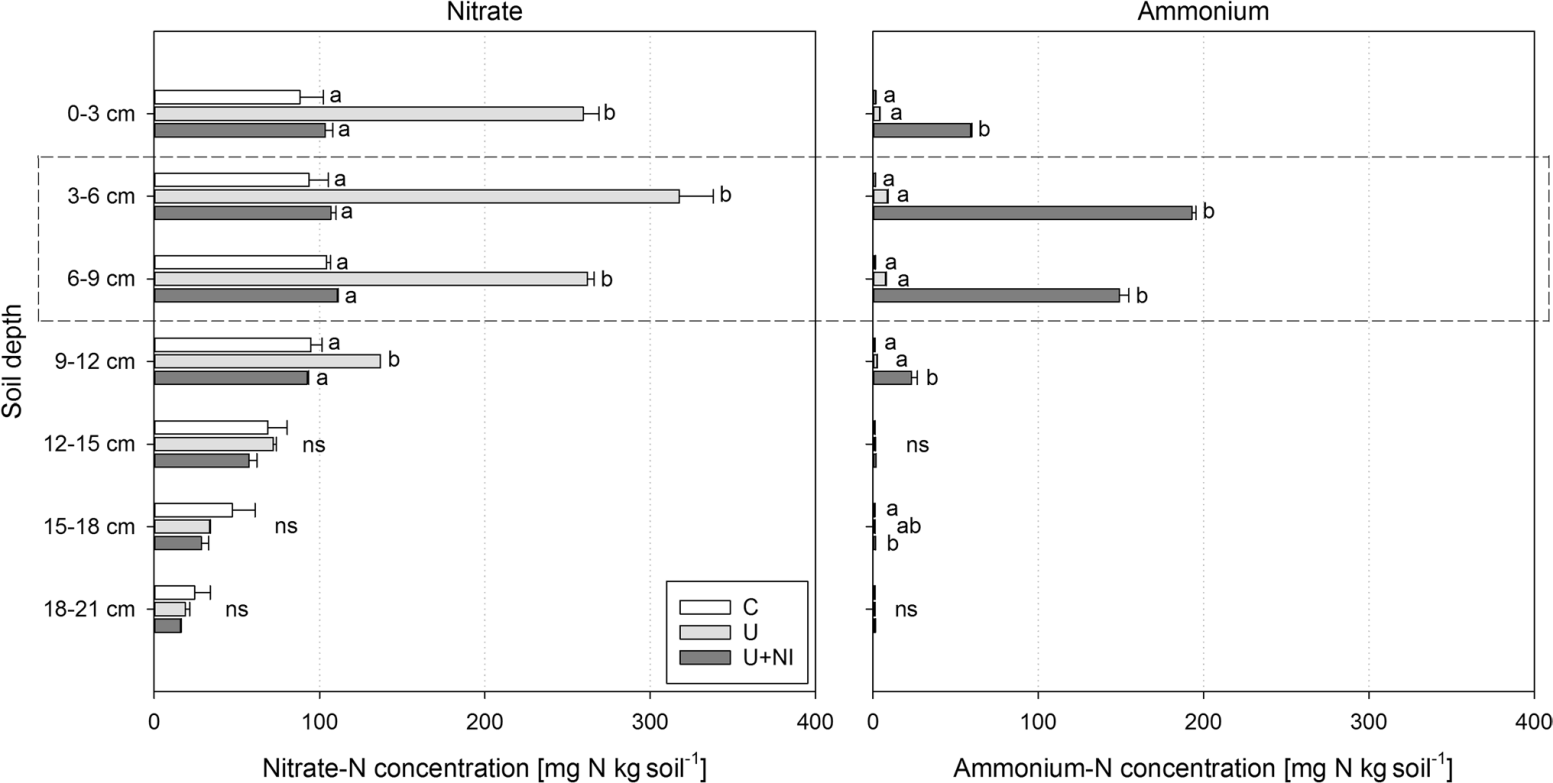
Depth distribution of readily available N and adsorbed N species NO_3_^−^ (left) and NH_4_^+^ (right) in unplanted soil columns (experiment 2) for the treatments control (“C”), urea (“U”) and urea with nitrification inhibitor (“U+NI”) on day 17, corresponding to 17 DAP in experiment 3. Significant differences between treatments within each layer (*p* < 0.05) are indicated by different letters, ns = not significant. Dashed box indicates area of fertiliser placement. Error bars indicate standard error.

Analysis of N species in the soil extract showed the distribution of mineral NO_3_^−^ and NH_4_^+^ within the soil columns, separately for layers with 3 cm in height. The maxima of both N forms were found in the layer in 3–6 cm depth, representing the fertiliser layer. Also the adjacent layers revealed higher concentrations than the remaining parts of the soil columns (Fig. 4).

NO_3_^−^ concentration in C and U+NI was almost identical and much lower than in U. In contrast, NH_4_^+^ concentration in C and U was similar and much lower than in U+NI. NO_3_^−^ was available in all treatments and all soil depths. NH_4_^+^ was more restricted to the fertiliser layer and only in U+NI considerable concentrations were detected. As for NO_3_^−^, significant and relevant differences for NH_4_^+^ concentration were found in soil layers down to a depth of 12 cm.

### Experiment 3: influence of plants on soil chemical conditions, plant growth development and root response of *Vicia faba* and *Hordeum vulgare* over time

For all treatments, initial pH values were statistically the same for planted (experiment 3) and unplanted samples (experiment 2), even though Fig. 2 reveals distinct differences. With time, differences started to develop within the treatments C and U+NI, depending on plant presence or absence. Planted samples tended to have higher pH values compared to their unplanted counterparts, but only for the treatment U+NI a difference at *p* < 0.05 was found (Fig. 2).

In C, the pH increased by about 0.9 units with plant presence within 16 days (*p* < 0.01). In U+NI, the pH increase was smaller (about 0.4 units, *p* < 0.01) compared to C. In contrast, the initial pH in the soil solution from U was the same as in U+NI but decreased about 1.2 units (*p* < 0.01) within 12 days of plant development. Both graphs for planted and unplanted samples in U showed a similar development.

NO_3_^−^ and NH_4_^+^ concentrations from the planted columns were very similar compared to the unplanted columns from experiment 2 (Fig. 3). No differences (*p* < 0.05) were found between planted and unplanted columns apart from U at 15 DAP, where the treatment with plant presence was already depleted, while 0.2 mM NH_4_^+^ was left in the unplanted counterpart (*p* < 0.05). In all planted treatments, NO_3_^−^ concentrations were greater at 15 DAP compared to − 1 DAP (*p* < 0.01). Decreasing NH_4_^+^ concentrations were found in U and U+NI at 15 DAP compared to − 1 DAP (*p* < 0.01).

#### Aboveground biomass and N-uptake by Vicia faba and Hordeum vulgare

**Figure 5:**
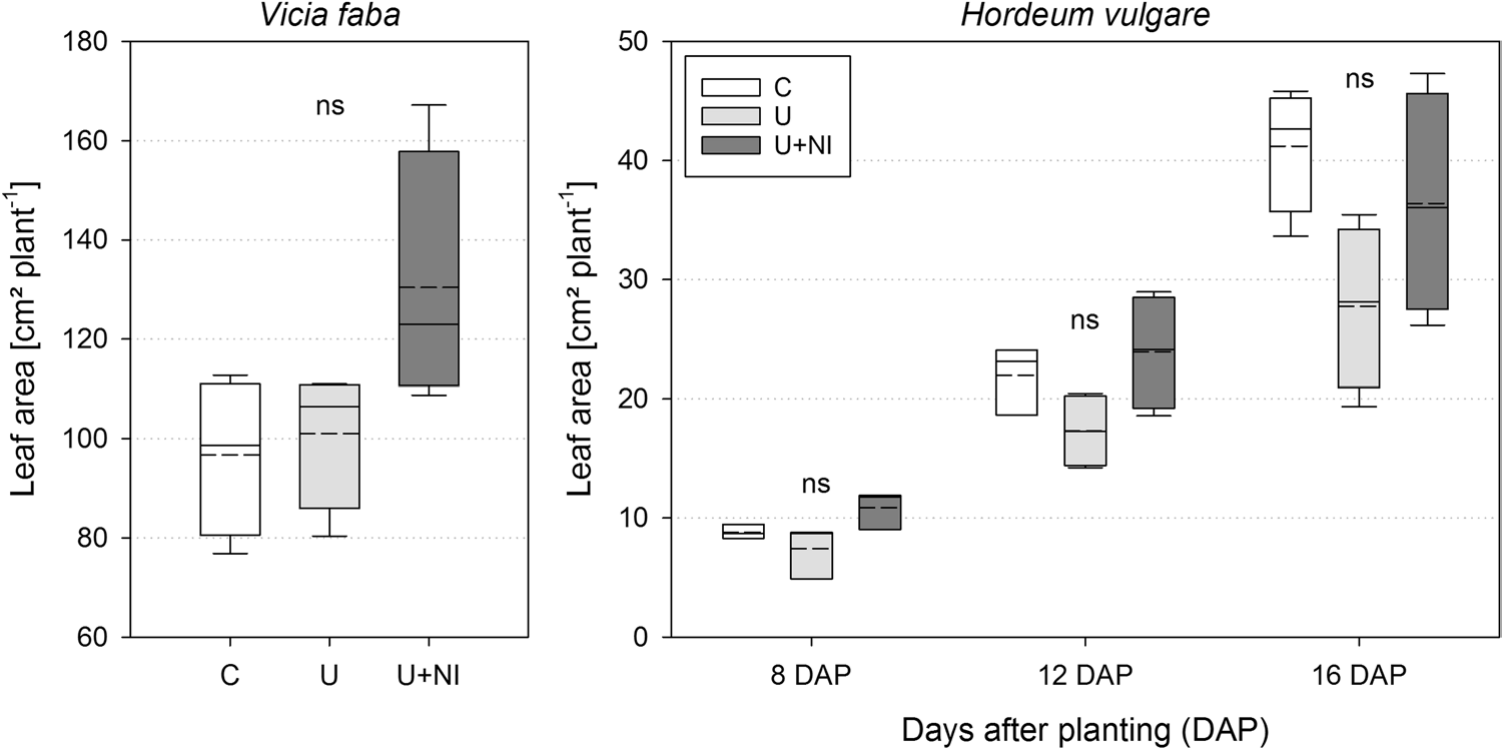
Leaf area for *Vicia faba* (left) after 16 days and for *Hordeum vulgare* (right) over time for the treatments control (“C”), urea (“U”) and urea with nitrification inhibitor (“U+NI”). Significant differences (*p* < 0.05) between treatments for each point in time are indicated by different letters, ns = not significant; dashed lines in the boxplots represent mean values while solid lines represent the median.

For *Vicia faba*, no significant differences were found between all treatments regarding leaf area (Fig. 5, left) and shoot fresh mass (Supplementary Fig. S5, left). A tendency towards larger values in U+NI was observed for leaf area compared to C (*p* < 0.1). N-concentration in the shoot of *Vicia faba* showed the same tendency as shoot mass and leaf area with the highest values in U+NI (Supplementary Fig. S6, left).

For *Hordeum vulgare*, leaf area was also not different for all treatments and all time steps (Fig. 5, right). Shoot fresh weight was affected by the different fertiliser treatments (Supplementary Fig. S5, right). After 16 days, mean shoot fresh mass in U was reduced by 42% compared to C (*p* < 0.05).

Similar results were found for shoot N concentration. Shoot N concentrations in *Vicia faba* and *Hordeum vulgare* ranged from 4.9 to 7.4% dry mass. For *Vicia faba*, no difference was found (Supplementary Fig. S6, left). For *Hordeum vulgare*, mean shoot N concentration on day 12 was 0.5% smaller (*p* < 0.05) in U compared to C and U+NI (Supplementary Fig. S6, right).

#### Root development

1. *Vicia faba*—root growth dynamics measured by X-ray CT and root growth analysis with WinRHIZO at the end of the experiment (17 DAP).

#### Visualisation of root growth development over time by X-ray CT and soil-root distances (Euclidian distance map transform)

**Figure 6:**
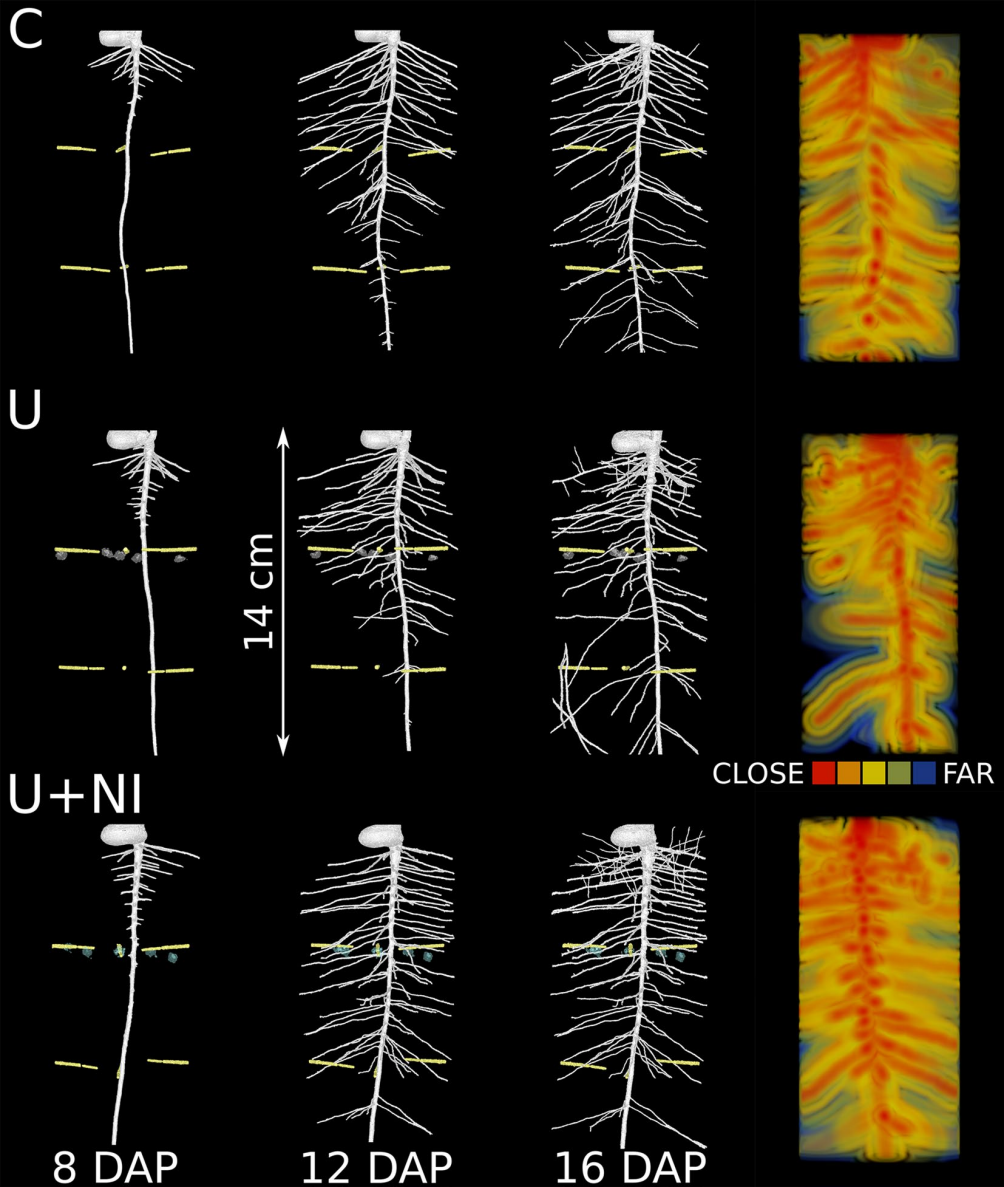
Time series of root growth development of *Vicia faba* for all three treatments (characteristic representatives for control (“C”), urea (“U”) and urea with nitrification inhibitor (“U+NI”) and visualisation of relative frequencies of soil-root-distances (representative layers) in 2 mm steps. Micro suction cups are represented in yellow. Remaining voids from fertiliser granules are indicated in grey (middle row) for urea and turquoise (bottom row) for urea with NI.

Examples of segmented root systems of *Vicia faba* are shown in Fig. 6 as time series from one representative replicate for each treatment. The most representative replicate was chosen, with individual root length closest to the mean value of the respective treatment. All other projections can be found in the supporting information (Supplementary Fig. S7).

#### Distance maps: relative frequencies of soil-root-distances

**Figure 7:**
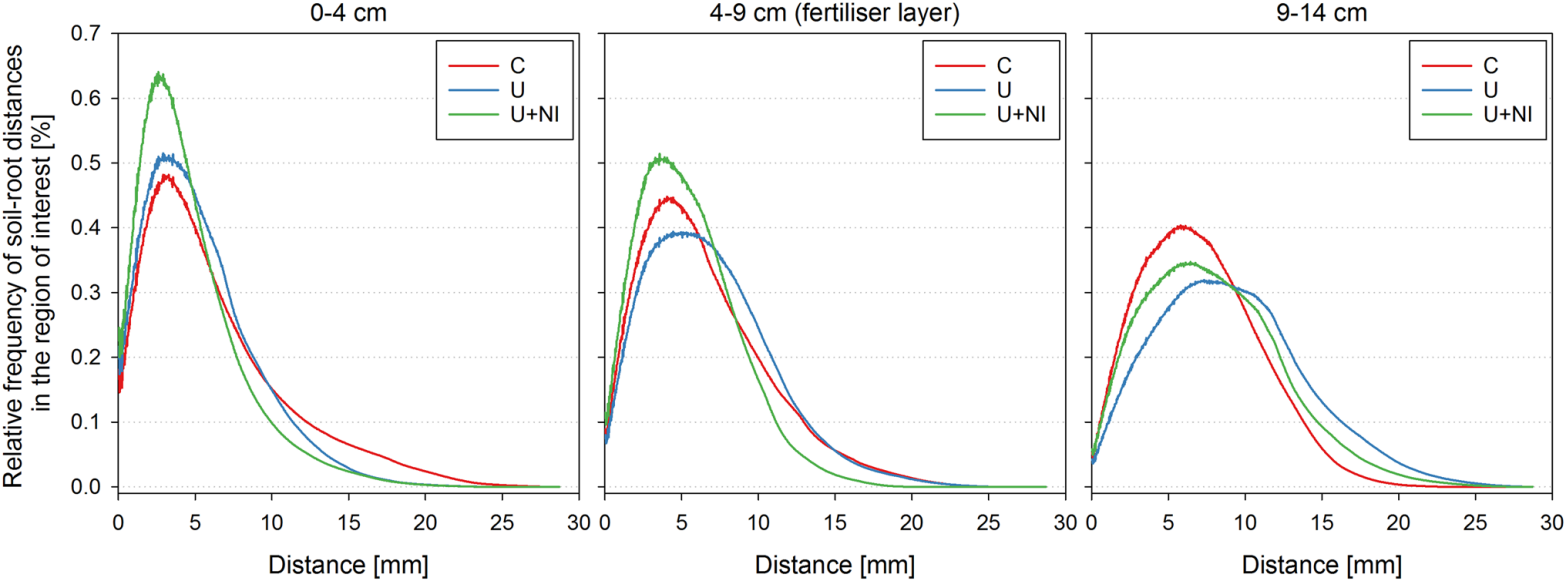
Relative frequencies [% of all soil voxels in the respective layer] of soil-root distances at 16 DAP for representative samples given in Fig. 6, separated in 3 layers, representing the fertiliser layer (middle) and both parts above (left) and below (right) the fertiliser layer. The control treatment (“C”) is given in red, urea (“U”) in blue and urea with nitrification inhibitor (“U+NI”) in green.

Information on the spatial extension of the root system and the three-dimensional exploration of the soil volume by the roots is insufficiently represented in the quantification of root lengths or number of roots. The true potential of 3D data is only revealed when geometric or topological measures are used for quantification. Relative frequencies of soil-root distances for the examples shown in Fig. 6 are given in Fig. 7 for the final state at 16 DAP for 3 layers. Presence of second order laterals in 0–4 cm compared to the other layers is reflected in a shift of the maximum to shorter distances. The larger number of second order laterals in the representative sample chosen for U+NI (Fig. 6) is reflected in the maximum for U+NI being higher than for the representative samples selected for C and U. The direct comparison of segmented CT root system architecture in Fig. 6 and the relative frequencies of soil-root distances illustrate how sensitive changes in root architecture can be captured by this new measure. Deriving relative frequencies of soil-root distance is less time consuming than deriving the number of second order laterals. Relative frequency curves for all individual samples can be found in the supporting information (Supplementary Fig. S8). From relative frequency distribution (RDH) the expectation value 〈RDH〉 (mean soil-root-distance) can be calculated. This value is closely related to the classical parameter half mean distance between roots (HMD) for equidistant distributions^56^. 〈RDH〉 values are provided in the supplement (Supplementary Fig. S9).

#### X-ray CT analysis

Root length and number of first and second order laterals showed no differences between treatments. This is true for all three points in time (8, 12 and 16 DAP) and soil depths (Supplementary Figs. S10–S13).

#### WinRHIZO analysis

Total root length as well as root length per functional diameter classes showed no differences between treatments (Supplementary Fig. S14).

2. *Hordeum vulgare* root growth dynamics analysed with WinRHIZO over time and in 3 separate soil layers.

**Figure 8:**
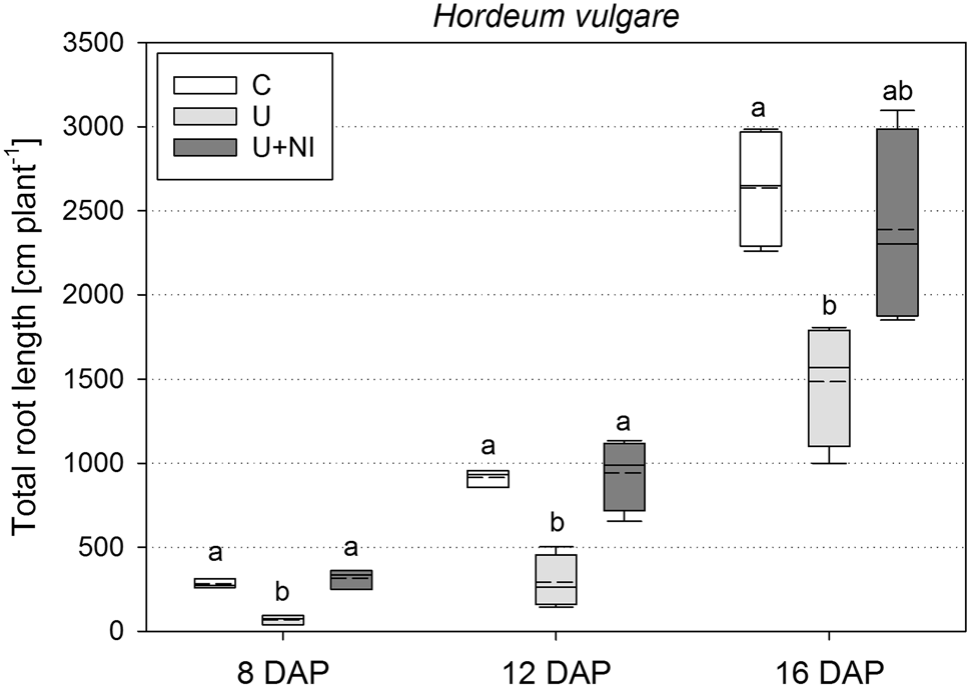
Total root length of *Hordeum vulgare* plants for the treatments control (“C”), urea (“U”) and urea with nitrification inhibitor (“U+NI”). Significant differences (*p* < 0.05) are indicated by different letters; dashed lines in the boxplots represent mean values while solid lines represent the median.

Total root length of *Hordeum vulgare* in U was considerably smaller compared to C and U+NI throughout all harvests (Fig. 8). Compared to C, mean total root length in U was 75% smaller at 8 DAP (*p* < 0.05), 68% at 12 DAP (*p* < 0.05) and 44% smaller at 16 DAP (*p* < 0.05). Because of large deviation, the difference of 902 cm in mean total root length between U and U+NI at 16 DAP was barely not significant (*p* = 0.06).

#### Root length per soil depth and diameter class

**Figure 9:**
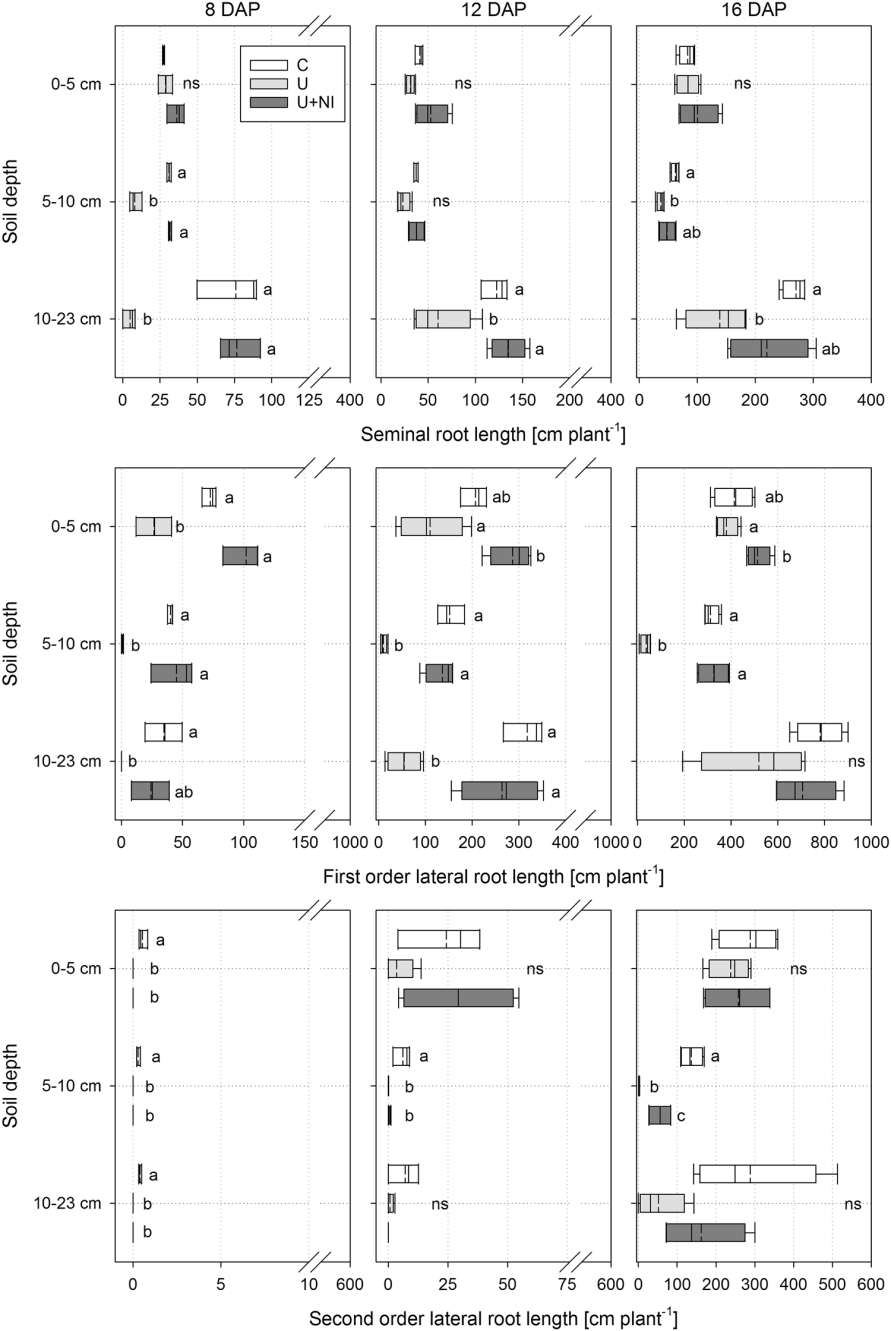
Root length of *Hordeum vulgare* plants, separated for three depths and root orders for each sampling point for the treatments control (“C”), urea (“U”) and urea with nitrification inhibitor (“U+NI”). Top layer (0–5 cm) is above fertiliser zone, bottom layer (10–23 cm) is below fertiliser zone. Top row = seminal roots, middle row = first order laterals, bottom row = second order laterals. Statistical comparison of root length is performed per layer between treatments. Significant differences (*p* < 0.05) are indicated by different letters; ns = no significant difference for *p* < 0.05; dashed lines in the boxplots represent mean values while solid lines represent the median.

The analysis of the root lengths per soil depth and functional diameter classes (Fig. 9) underlined the results of the total root length (Fig. 8). Furthermore, it becomes apparent that the inhibition of root growth in U took place particularly in the area of local fertilisation. This applies to all root diameter classes, namely seminals (upper row), first order laterals (middle row) and second order laterals (bottom row). For second order laterals this is only the case at 16 DAP, where the smallest length was found in U (1.4 cm), followed by U+NI (51 cm). The root length of both treatments was much smaller (*p* < 0.05) compared to C (136 cm).

There was no influence on the seminal roots observed above the fertiliser layer. After 8 days, the length of first order laterals in U was only 63% of the mean length in C (*p* < 0.05), but this difference disappeared over time. After 8 days, second order laterals were found only in C. The treatments did not differ from each other later on.

Below the fertiliser layer, clear differences were found regarding the length of seminal roots and first order laterals. The latter were almost non-existent after 8 days in this layer (< 1 cm). This difference became smaller over time and was no longer detectable after 16 days (*p* > 0.05).

#### Number of first order laterals

**Figure 10:**
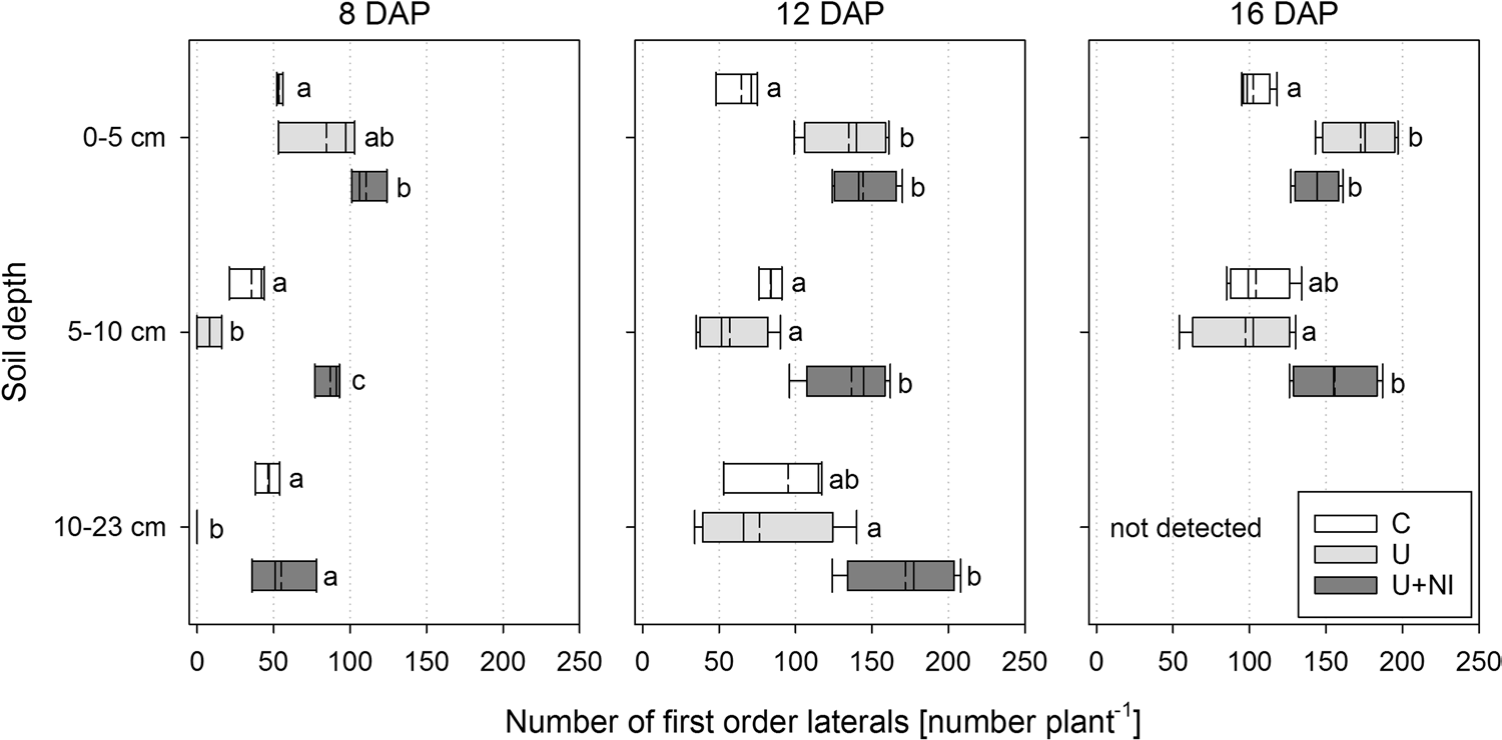
Number of first order lateral roots of *Hordeum vulgare* plants for treatments control (“C”), urea (“U”) and urea with nitrification inhibitor (“U+NI”), separated for three depths for each harvest. Top layer (0–5 cm) is above fertiliser zone, bottom layer (10–23 cm) is below fertiliser zone. Statistical comparison is performed for number of first order laterals per layer between treatments. Significant differences (*p* < 0.05) are indicated by different letters. Bottom layer at 16 DAP was too complex for a reliable analysis; dashed lines in the boxplots represent mean values while solid lines represent the median.

Length of laterals was not significantly different between U+NI and C (Figs. 8, 9), but morphologic appearance of the root systems showed differences. Number of first order laterals was highest in the U+NI treatment. This is the case for almost all sampling dates and soil depths and especially in the fertiliser layer (Fig. 10). A very distinct difference was present as early as 8 DAP (Fig. 10, left). Whereas only 8 (± 5) first order laterals were counted on average in U, 36 (± 8) first order laterals were found in C and 87 (± 5) in U+NI (*p* < 0.05).

## Discussion

### Methodological approach

The aim of the experimental setup was to study the local impact of NH_4_^+^ and NO_3_^−^ on root architecture in a similar way as in the classical work of Drew^26^, but simulated by the application of urea fertiliser with and without a nitrification inhibitor under field relevant soil conditions. The limitations for soil studies which Drew^26^ faced at his time, i.e. to follow soil solution N speciation dynamics over time and simultaneously observe the response of root morphology in situ, were overcome in the present study by soil solution sampling with micro suction cups and X-ray CT scanning associated with detailed, supervised image analyses.

The temporal and spatial resolution chosen for soil solution collection was sufficient to illustrate the change in NH_4_^+^ to NO_3_^−^ ratio and absolute concentrations over time as well as the change in pH associated with N transformation in soil and plant N assimilation. Although the absolute numbers are smaller, similar tendencies for N-dynamics were found in a field study by Kirschke et al.^60^, using similar types of urea fertilisers. The large discrepancies between NH_4_^+^ and NO_3_^−^ ratios in soil solution compared to soil extraction nicely illustrate the problem of transferring concentration related results from hydroponics and agar plates to soil systems and will be discussed in more detail below.

By splitting the watering between top and bottom we avoided NO_3_^−^ leaching. Background NO_3_^−^ from mineralisation of organic matter was moved with irrigation water to the fertiliser layer (treatment C in Fig. 4). In U+NI, NH_4_^+^ was indeed the dominant N form in soil extracts (KCl), but it largely reflects the fraction adsorbed to the soil matrix. By contrast, in soil solution NO_3_^−^ dominated, although concentrations of NH_4_^+^ were substantial and much higher in NI treatments than in U treatments without NI (Fig. 3). This largely results from the fact that the mobility of NO_3_^−^ is considerably higher than that of NH_4_^+^, since the latter is adsorbed to clay minerals and organic matter. Therefore, a much larger proportion of NO_3_^−^ (even when based on nitrification of NH_4_^+^) is found in the soil solution compared to NH_4_^+^. In this context. NO_3_^−^ production can be basically considered as a two-step reaction, first desorption of adsorbed NH_4_^+^ into the solution and second, nitrification of dissolved NH_4_^+^ to NO_3_^−^. Under optimal conditions nitrification in soil is a rather quick process. NH_4_^+^ concentration in soil solution would be constant over time as long as the desorption rate (i.e. NH_4_^+^ supply to the solution) is equal to the consumption rate (i.e. NO_3_^−^ production by nitrification of dissolved NH_4_^+^). This means that NH_4_^+^ concentration in solution remains constant over time, while NO_3_^−^ concentration increases significantly. In the case of this study, NH_4_^+^ concentration in U decreased, probably because the nitrification rate exceeded the NH_4_^+^ desorption rate. In U+NI nitrification was considerably limited for a while (as visible in the missing NO_3_^−^ production in Fig. 3) and hence, NH_4_^+^ is only removed from the solution towards the end of the experiment.

This is also reflected in the change of soil solution pH with time in the fertiliser layer (Fig. 2). In U+NI, plant N nutrition was a mixture of NH_4_^+^ assimilation originating from the fertiliser and assimilation of background NO_3_^−^ from the soil. Both processes have an influence on pH due to release or consumption of H^+^ or OH^−^, respectively. In the control (“C”), NO_3_^−^ nutrition of plants caused a pH increase in the planted treatment (Experiment 3; Fig. 2). In contrast, active soil NH_4_^+^ turnover in the U treatment had a stronger impact on soil solution pH than plant uptake. This revealed the strong impact of nitrification and the associated release of protons on soil solution pH. When NH_4_^+^ turnover was actively inhibited by additional NI application (treatment U+NI) soil solution pH also increased due to plant uptake of sufficently available native NO_3_^−^ in the soil.

Regarding root growth, we expected increased root length in U and an increased initiation of lateral roots in U+NI as a response to higher NO_3_^−^ and NH_4_^+^ concentrations, respectively—and especially in the layer of fertiliser placement. In part, this is the case and our results are in line with those from Drew^26^, but only in terms of higher initiation of laterals due to NH_4_^+^ in *Hordeum vulgare* (‘Marthe’). In contrast, we have not observed an increase in root length due to NO_3_^−^, but instead inhibition of root elongation in *Hordeum vulgare* (‘Marthe’).

The analysis of the relative frequency distribution of root-soil distances, based on Schlüter et al.^56^ was able to reveal small differences in root architecture (Figs. 6, 7). To further exploit this tool the approach of Schlüter et al.^56^ to fit a triangular gamma model with just four parameters has to be developed further. This will then enable a statistical evaluation of changes in the shape of the distribution function. The latter is not captured by just deriving the mean root-soil distance.

The less pronounced response to NH_4_^+^ in our experiment and an even negative one to NO_3_^−^ in *Hordeum vulgare* (‘Marthe’) may be explained by (i) differences in absolute concentrations, (ii) differences in NO_3_^−^: NH_4_^+^ ratio, (iii) uncertainty about the impact of adsorbed NH_4_^+^, (iv) differences in temporal development of NO_3_^−^ and NH_4_^+^ concentrations, (v) ratio of N placement volume to total volume (vi) N-status of control treatment and (vii) species preference for NH_4_^+^ or NO_3_^−^ and susceptibility to NH_4_^+^ toxicity or inhibition by high NO_3_^−^.

As stated by Nacry et al.^9^, a typical root growth response to N that is applicable for all species and conditions is almost impossible to define, as many biotic and abiotic factors are responsible for the final shaping of the root system architecture. Nacry et al.^9^ highlighted two very general aspects that hold true for most circumstances: (1) high N status (in root and/or shoot) leads to a systemic repression of lateral root growth and (2) exogenous NO_3_^−^ or NH_4_^+^ can have local effects on root growth.

The N status of the shoots was high but not exceedingly high given the very young age of the plants^61^. Exogenous concentration of NO_3_^−^ and NH_4_^+^ altered root growth during the early plant growth stage investigated. It is possible—in fact likely—that later season growth differs as plant demand changes with time.

### Root growth response in different systems

Different systems of plant cultivation range from agar plates to nutrient solution to soil and may vary greatly in their response of root growth. Very pronounced root growth responses were found in studies using agar plates with extremely controlled conditions for growth of *Arabidposis*^21–25^. These approaches reveal the potential of root growth response to different N-forms and concentrations. But the utilised distributions and concentrations on agar are not realistic for soils, as applied concentrations in these studies are typically low and constant, and the pH is buffered to exclude potential influences. In these systems, laboratory chemicals are used instead of fertilisers and microbial activity is not present, thus preventing oxidation of ammonium to nitrate. This also means that there are no dynamics regarding the concentration and ratio of the two N-forms in these systems.

An optimum curve for intermediate N-concentrations of both N-forms was determined in several studies, with lower and higher concentrations leading to smaller total root length, mainly explained by the negative response of lateral root growth^21,22,25^. Zhang et al.^25^ found a strong inhibition of lateral root growth of 50% for 50 mM NO_3_^−^. The described reaction of *Arabidopsis* with presence of many but very short laterals is very similar to what we observed for *Hordeum vulgare* (‘Marthe’) in the fertiliser layer in the U treatment with even higher NO_3_^−^ concentrations up to 100 mM (see Supplementary Fig. S15). Though, we did not observe the same for *Vicia faba* (‘Fuego’)*.*

The problem of transferability similarly accounts for nutrient solution studies^10–20^. Typically, applied concentrations are rather low, absorption is irrelevant and chemical and nutritional conditions are kept constant by adding buffers and replacing the solution in a high frequency. Moreover, in solution nutrient mobility is high and a large solution volume is in direct contact with the root system, which is not the case under natural soil conditions.

Similar to results from agar plates, there is evidence in these studies for a growth stimulating effect at low NO_3_^−^ concentrations, while higher concentrations result in growth inhibition^17,20^. Other studies have also found that at low concentrations of both N-forms, NH_4_^+^ can be more beneficial for root growth than NO_3_^−^_,_ e.g. for potato in Gerendás and Sattelmacher^13^ or maize in Bloom et al.^11^ and the citations therein. In contrast, regarding higher concentrations, NH_4_^+^ impeded root growth more than NO_3_^−^, e.g. for maize on 4 mM^62^ or tomato on 8–10 mM^63,64^.

These results emphasise that plant species and even genotypes of the same species can be quite different regarding their susceptibility against N-form inhibition or even toxicity, or the other way round, their preferred N-form and concentration. This can also be derived from our data, as *Vicia faba* (‘Fuego’) was not distinctly inhibited by high NO_3_^−^, while lateral root length was markedly reduced in *Hordeum vulgare* (‘Marthe’).

These results illustrate well the fact that plant’s responses to localised nutrients fall on a spectrum^65^. Drew’s barley plants were definitely at the upper end of responsiveness, albeit under contrived laboratory conditions. Other species and experimental setups usually produce small, zero, or even, as here, negative responses. Not all experiments with localised nutrient availability yield distinct results^40,41^, and *Vicia faba* seems to be a relatively unresponsive species in general^46,66^.

Studies in soil, conducting pot experiments^35,36,38–45^ or field studies^11,15,16,37^ found more diverse results of plant growth response to different N applications, ranging from no effect to stimulation of root growth to toxicity. These differences are related to the fact that soils can have an extremely wide range of physical, chemical and biological conditions for plant growth compared to well-controlled conditions on agar plates or in nutrient solution. It should be borne in mind that results obtained from experiments carried out with soil are specific to the respective soils and therefore the soil properties must be taken into account when interpreting the results.

To overcome these given uncertainties, we monitored both—N-status and pH in soil solution as well as root growth development—concurrently. By introducing the 3 week phase of incubation, reasonable N-input rates for pot experiments, together with a sufficient spacing between the urea granules themselves and to the seeds, we avoided NH_4_^+^ toxicity that was found in Xu et al.^38^ and Pan et al.^37^, as well as for the highest N-application rates in Anghinoni et al.^36^ and Anghinoni and Barber^35^.

Apart from the local root growth depression by high NH_4_^+^, Xu et al.^38^ found higher total root length in the treatment with local urea application without NI in comparison to the control without N input and to the +NI treatment. Similar to our results for *Vicia faba*, this cannot be explained by a local root foraging into the fertiliser patch, as the gain in root length was achieved in areas further away from the fertiliser in Xu et al.^38^. But one has to note that in Xu et al.^38^, NH_4_^+^ in soil extract from the fertiliser placement was very high in both treatments, almost twice the NH_4_^+^ concentration in our study, so that their final result of root growth distribution may be a consequence of both, stimulation by NO_3_^−^ and inhibition by NH_4_^+^.

In contrast, Anghinoni et al.^36^ and Anghinoni and Barber^35^ measured N-concentration in soil solution at the end of the growth experiment. They observed highest dry weight of shoots and roots for maize at a concentration of 1.33 mM NH_4_^+^ in soil solution, while higher NH_4_^+^ markedly reduced plant growth. In our study, both treatments C and U+NI are characterised by the same NO_3_^−^ conditions but additional NH_4_^+^ in U+NI. Hence, differences between those two treatments can be attributed to NH_4_^+^. We found NH_4_^+^ concentrations in soil solution higher than 1.33 mM, locally in the fertiliser layer, but we have not observed toxicity symptoms even though Fabaceae and barley have been assigned to be NH_4_^+^ sensitive^67^. Genotypes of the same species may differ in the intensity of their response to certain stimuli. For *Vicia faba* and *Hordeum vulgare* this is known for e.g. salt stress^68,69^ and drought tolerance^70,71^. Similar studies on root growth as a function of N-form and concentration for a number of genotypes would be of great benefit.

Anghinoni et al.^36^ cite further studies that emphasise the relevance of sorption, as 1 mM in sand culture was toxic to tomato^72^ and muskmelon^73^, while NH_4_^+^ toxicity was not observed in two corn genotypes grown in vermiculite with up to 25 mM NH_4_^+^^74^. Hence, sorption but also the soil specific dynamic equilibrium between absorbed and NH_4_^+^ in solution needs to be taken into account to evaluate the potential for root growth responses to external NH_4_^+^, NO_3_^−^ and the ratio of both N-forms. As shown here and recently reported by Kirschke et al.^60^ the soil solution concentration of NH_4_^+^ is maintained at a considerable level by NI application, highlighting nitrification as a key process of in situ NH_4_^+^ concentration and thus, suggest a possible interrelation of soil nitrification rates and root architecture.

## Conclusion

The combination of chemical analysis of soil solution and simultaneous analysis of root growth over time can provide important insights into the interaction between N fertiliser turnover, soil solution chemistry and the reaction of root growth to these conditions in situ. The use of non-invasive imaging techniques such as X-ray CT is of particular interest as it allows the explicit and time resolved quantification of the exploration of the soil volume by the growing root system in terms of soil-root-distances.

Plant species with contrasting root system architecture react differently to the given experimental conditions. While *Vicia faba* (‘Fuego’) shows hardly any response, *Hordeum vulgare* (‘Marthe’) displays root growth inhibition in response to high NO_3_^−^ concentration in the soil and a greater number of first order lateral roots due to higher NH_4_^+^ concentration. In contrast to artificial growth media such as agar and nutrient solution, a variety of biotic and abiotic factors like adsorption of NH_4_^+^ and microbial activity interact in a real agricultural soil system, which can strongly influence the root growth response. This underlines the difficulty of the transferability of results between different growth media and provides an indication of why results in soil are often different and less clear than in artificial media.

Further studies are necessary to investigate the influence of these factors and their interactions on root growth morphology in real agricultural soils. For an even more complete understanding of the plant response to the local availability of different N-forms, the physiological plasticity should be studied simultaneously with the root growth plasticity. Additionally, obtaining comparable data for other soils and plants is important for a better understanding of the soil–plant interaction related to N-nutrition on a larger scale. Furthermore, the comparison of several genotypes of the same species would be of great interest, in particular with respect to breeding for N use efficiency related to fertilisation.

## Supplementary information

Supplementary file 1.
