# Supplementary material for: Dynamics of localised nitrogen supply and relevance for root growth of Vicia faba (‘Fuego’) and Hordeum vulgare (‘Marthe’) in soil
Source: Sci Rep. 2020 Sep 25;10:15776. doi: 10.1038/s41598-020-72140-1 (PMC7519116; doi:10.1038/s41598-020-72140-1)
Supplement: Supplementary file 1 [file 41598_2020_72140_MOESM1_ESM.docx]

Supplementary information about the article

„Dynamics of localised nitrogen supply and relevance for root growth of *Vicia faba* (‘Fuego’) and *Hordeum vulgare* (’Marthe’) in soil”

Sebastian R.G.A. Blaser^1^, Nicolai Koebernick^2^, Oliver Spott^3^, Enrico Thiel^3^ & Doris Vetterlein^1,2^

^1^Helmholtz-Centre for Environmental Research GmbH – UFZ; Department of Soil System Science, Theodor-Lieser-Str.4, 06120 Halle (Saale), Germany

^2^Martin-Luther-University Halle-Wittenberg, Institute of Agricultural and Nutritional Sciences, Von-Seckendorff-Platz 3, 06120 Halle (Saale), Germany

^3^Agricultural Application Research, SKW Piesteritz GmbH, Am Wieseneck 7, 04451 Cunnersdorf, Germany

Author for correspondence: Sebastian Blaser

E-Mail: [sebastian.blaser@ufz.de](mailto:sebastian.blaser@ufz.de)

Content:

- Fig. S1: Temporal change of NO_3_^-^ concentration under standardised conditions in experiment 1
- Fig. S2: Change of soil solution pH with time 5 cm below the fertiliser layer
- Fig. S3: Temporal change of NO_3_^-^ and NH_4_^+^ concentrations in soil solution 5 cm below the fertiliser layer
- Fig. S4: Temporal change of osmotic potential in soil solution in both layers
- Fig. S5: Shoot fresh mass for *Vicia faba* and for *Hordeum vulgare*
- Fig. S6: Shoot N concentration for *Vicia faba* and for *Hordeum vulgare*
- Fig. S7: All visualisation steps of root growth development of *Vicia faba*, acquired by X-ray CT
- Fig. S8: All relative frequencies of soil-root-distances at 16 DAP, separated in 3 layers, representing the fertiliser layer as well as the layers above and below
- Fig. S9: Mean soil-root-distances at 16 DAP, separated in 3 layers, representing the fertiliser layer as well as the layers above and below
- Fig. S10: Tap and first order lateral root growth development of *Vicia faba* over time, acquired by X-ray CT
- Fig. S11: Length and number of second order lateral roots of *Vicia faba*, acquired by X-ray CT
- Fig. S12: Depth distribution of number of first order laterals from *Vicia faba* after 16 days, acquired by X-ray CT
- Fig. S13: Depth distribution of first order lateral root length from *Vicia faba* after 16 days, acquired by X-ray CT
- Fig. S14: Total root length and root length per functional diameter classes for *Vicia faba* after 16 days, acquired by WinRHIZO analysis
- Fig. S15: Example of root growth inhibition by high NO_3_^-^ in barley. Scanned roots in the fertiliser layer in the treatment U, 16 days after planting.


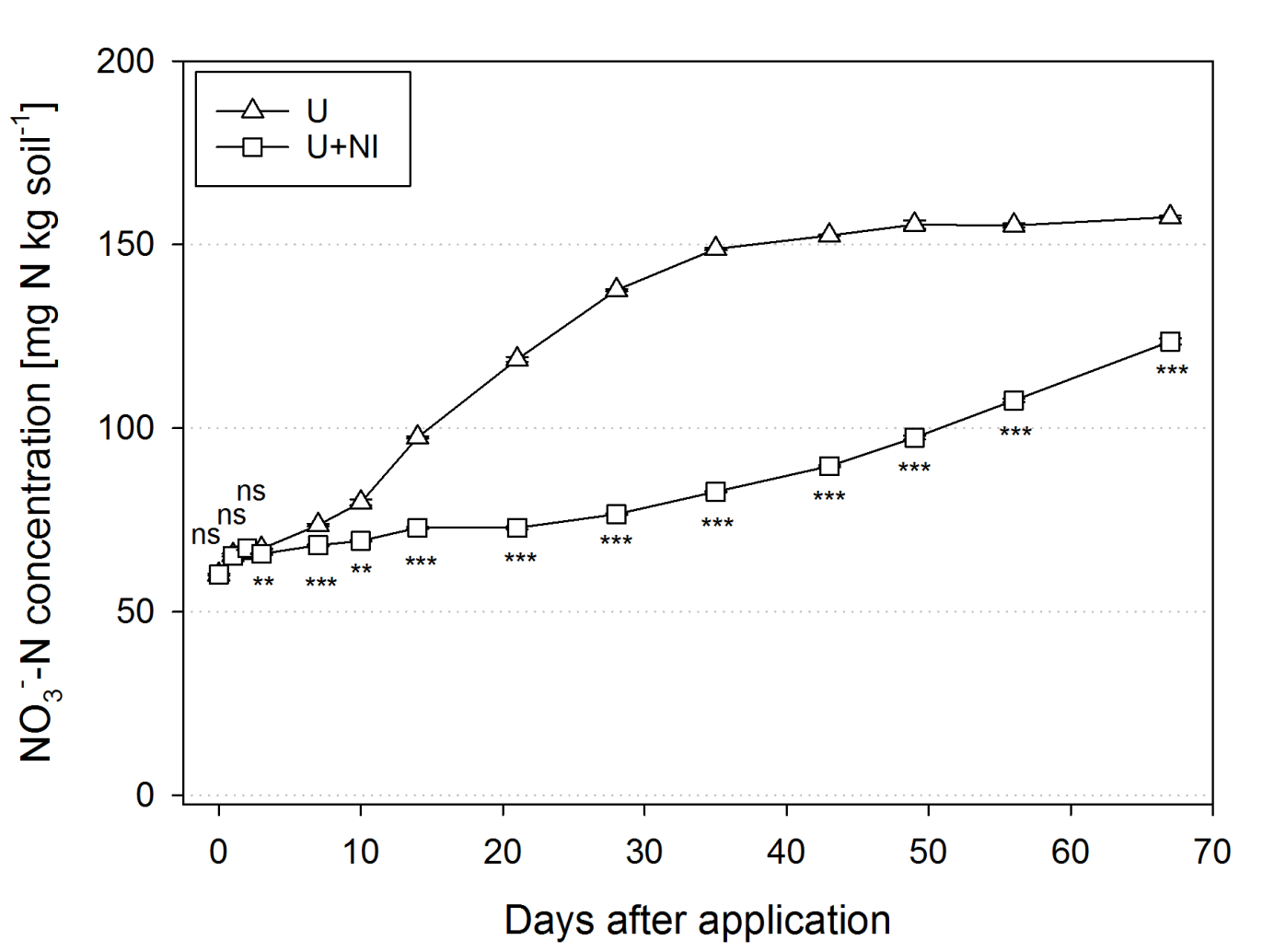


Figure S1: Temporal change of NO_3_^-^-N concentration under standardised conditions in experiment 1 for treatments urea (“U”, triangle) and urea with nitrification inhibitor (“U+NI”, square) in soil extract (1 M KCl). Asterisk indicates significant difference between treatments at *p*<0.05 (*), *p*<0.01 (**) and *p*<0.001 (***), ns = not significant. Error bars are masked by treatment symbols in most cases.


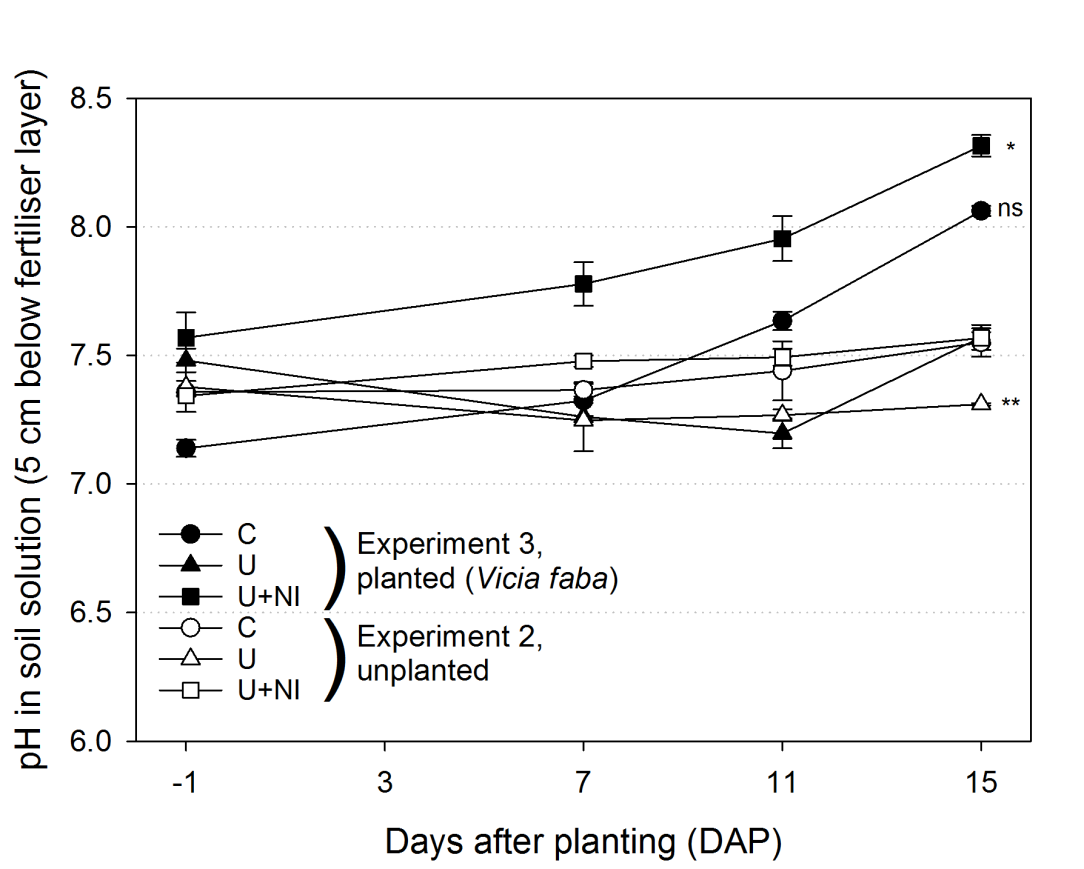


Figure S2: Change of soil solution pH with time 5 cm below the fertiliser layer in experiments 2 (open symbols) and 3 (closed symbols) with *Vicia faba* for treatments control (“C”, circles), urea (“U”, triangles) and urea with nitrification inhibitor (“U+NI”, squares). First sampling point (-1 DAP) defines starting conditions for root growth in experiment 3. Asterisk indicates significant difference between planted and unplanted treatments at *p*<0.01 (**), ns = not significant. Error bars indicate standard error.


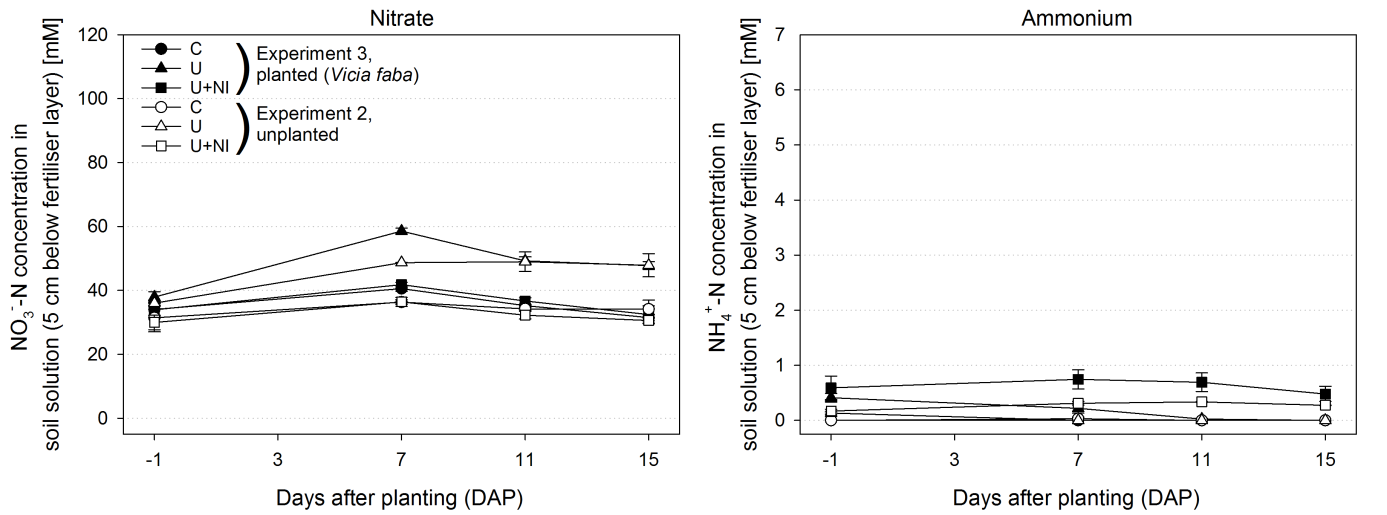


Figure S3: Temporal change of NO_3_^-^ (left) and NH_4_^+^ (right) concentrations in soil solution 5 cm below the fertiliser layer from experiments 2 and 3 with *Vicia faba* after three weeks of incubation for treatments control (“C”, circles), urea (“U”, triangles) and urea with nitrification inhibitor (“U+NI”, squares). Filled symbols indicate plant presence (experiment 3), open symbols indicate plant absence (experiment 2). Starting point is one day before plants were introduced in experiment 3.


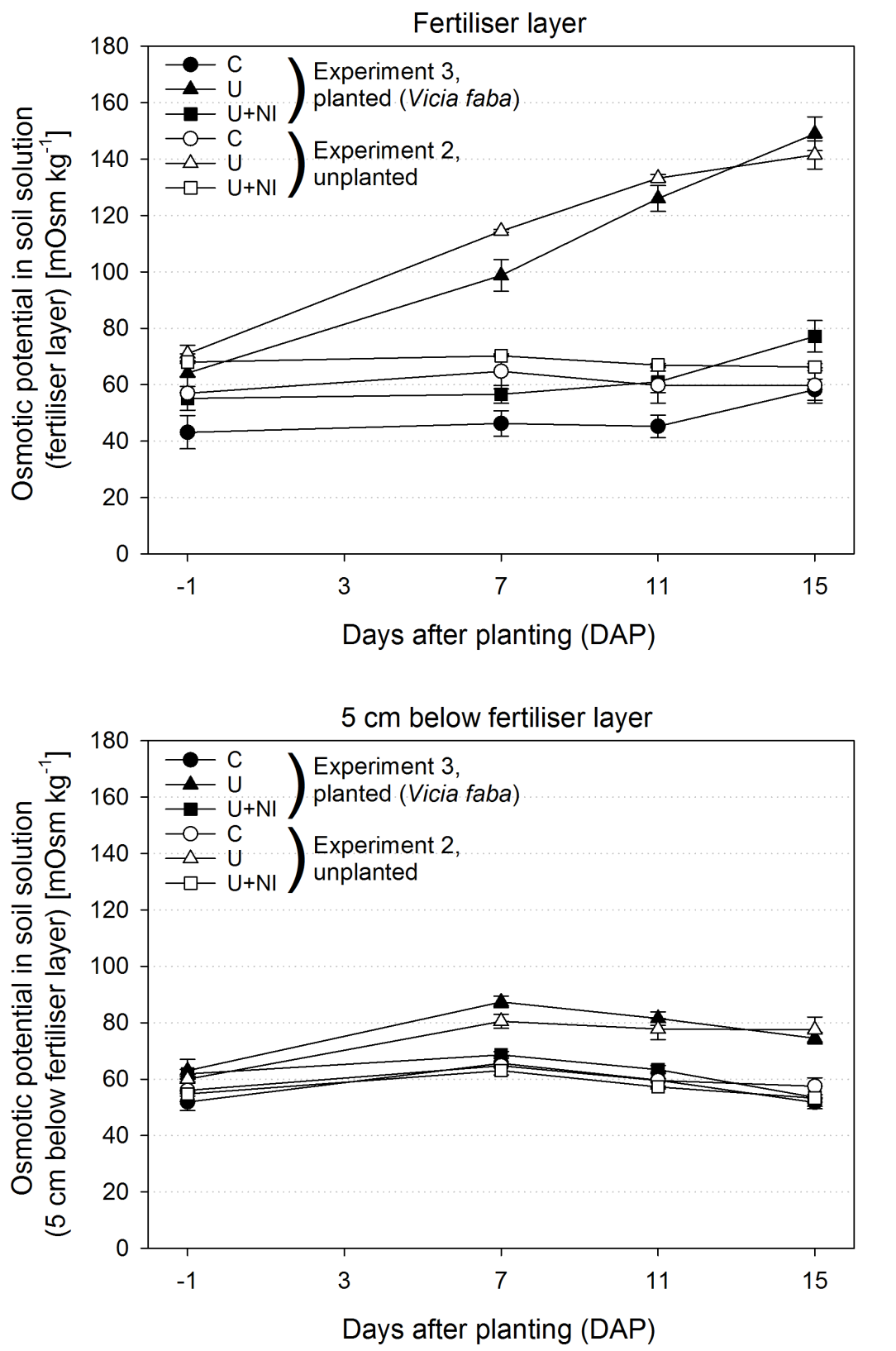


Figure S4: Temporal change of osmotic potential in soil solution in both layers from experiments 2 and 3 with *Vicia faba* after three weeks of incubation for treatments control (“C”, circles), urea (“U”, triangles) and urea with nitrification inhibitor (“U+NI”, squares). Filled symbols indicate plant presence (experiment 3), open symbols indicate plant absence (experiment 2). Starting point is one day before plants were introduced in experiment 3.


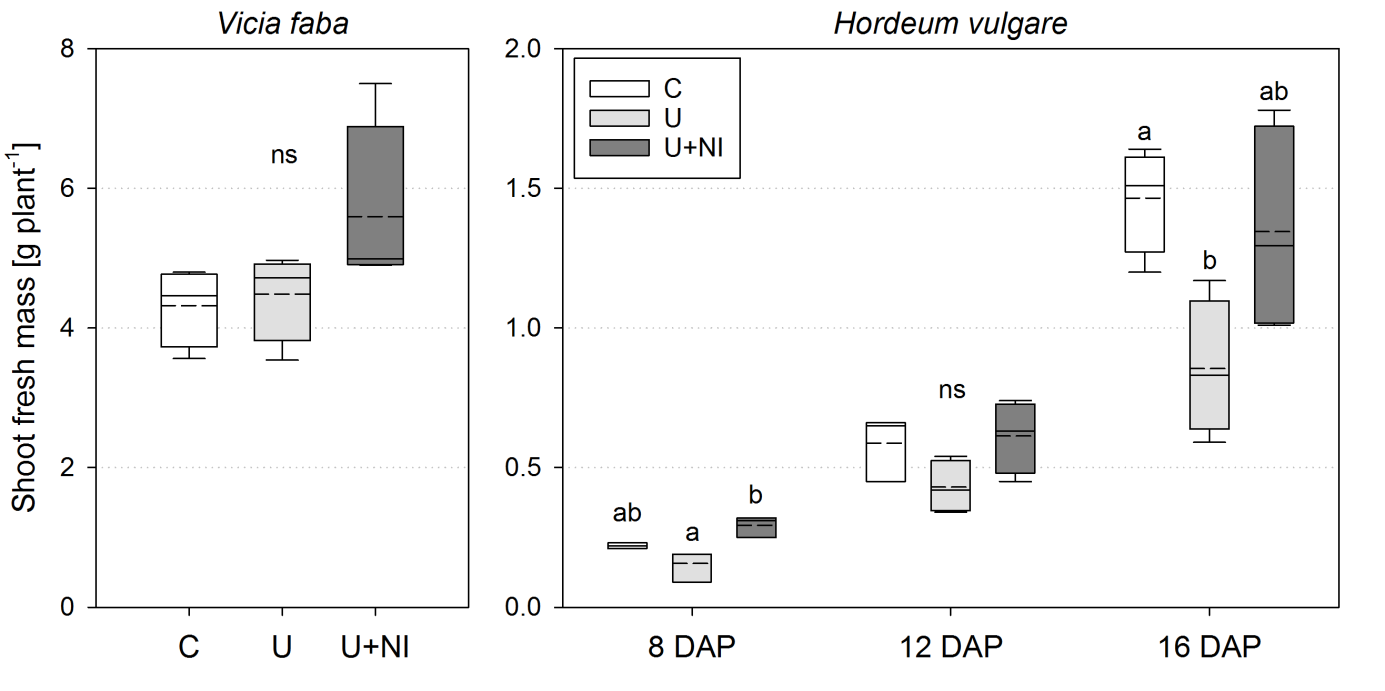


Figure S5: Shoot fresh mass for *Vicia faba* (left) after 16 days and for *Hordeum vulgare* (right) over time for the treatments control (“C”), urea (“U”) and urea with nitrification inhibitor (“U+NI”). Significant differences (*p*<0.05) between treatments are indicated by different letters, ns = not significant; dashed lines in the boxplots represent mean values while solid lines represent the median.


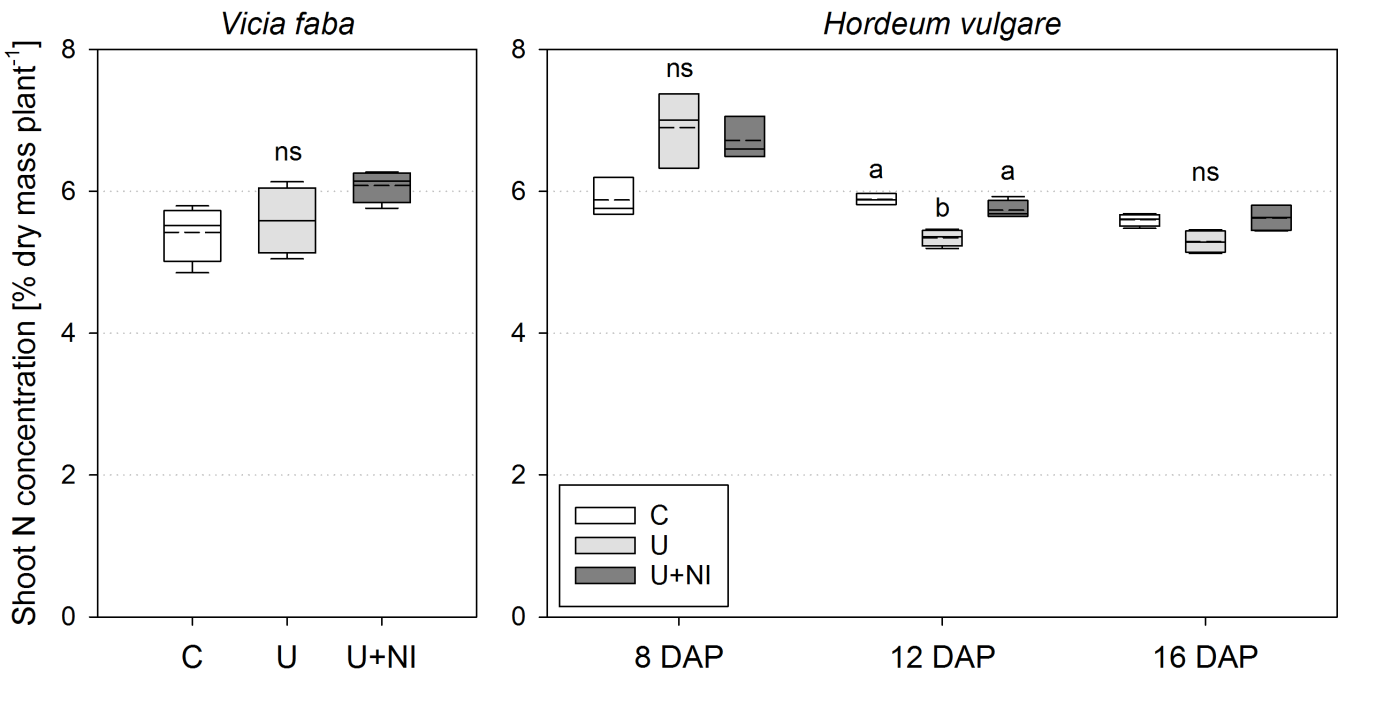


Figure S6: Shoot N concentration for *Vicia faba* (left) and *Hordeum vulgare* (right) in percent per dry mass per plant for the treatments control (“C”), urea (“U”) and urea with nitrification inhibitor (“U+NI”). Significant differences between treatments (*p*<0.05) are indicated by different letters, ns = not significant; dashed lines in the boxplots represent mean values while solid lines represent the median.


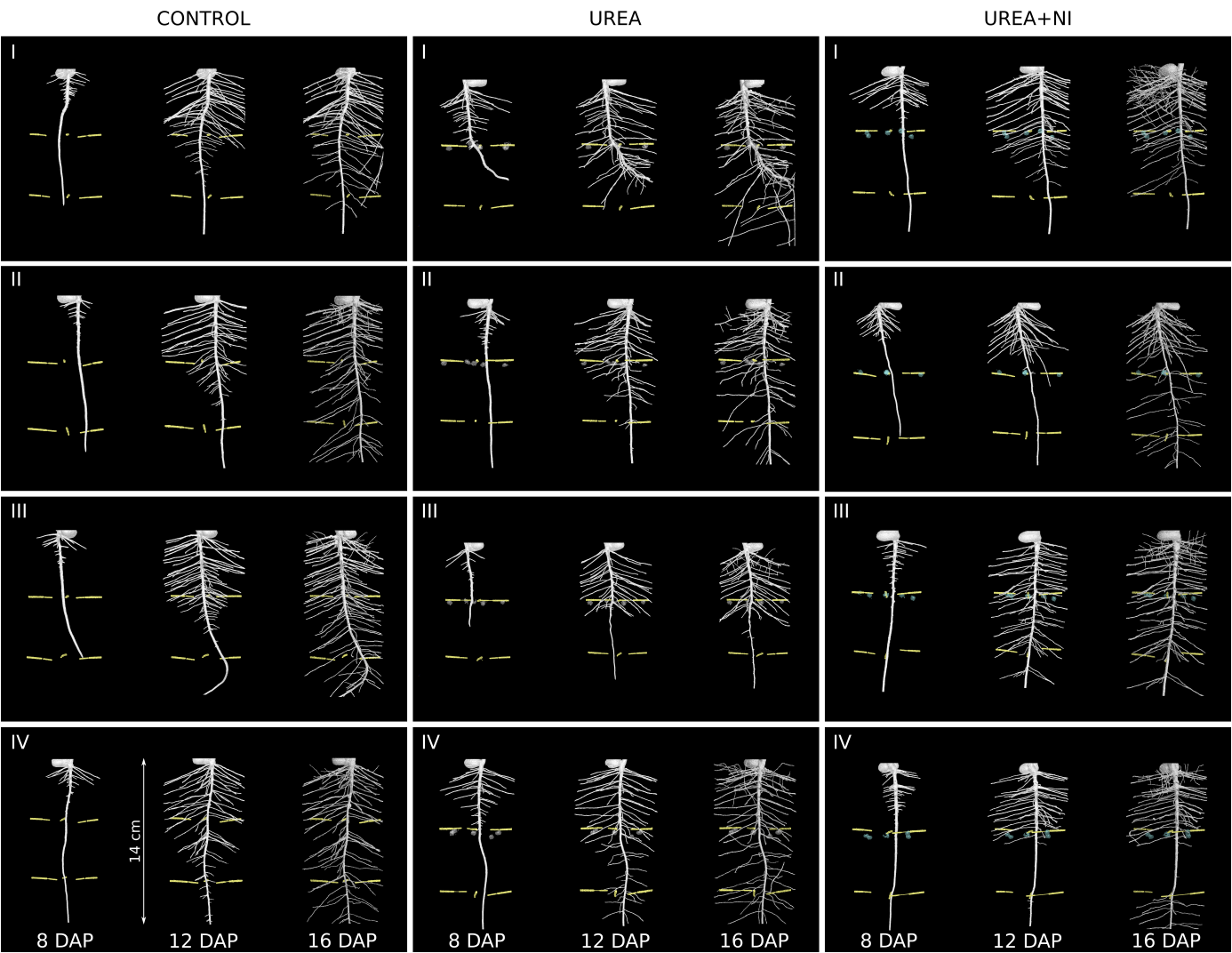


Figure S7: All steps of root growth development of *Vicia faba*, acquired by X-ray CT. Shown are all replicates (I-IV) for all treatments over time (8, 12 and 16 DAP). Micro suction cups are shown in yellow, fertilizer granules in grey (treatment urea) or turquoise (treatment urea with nitrification inhibitor).


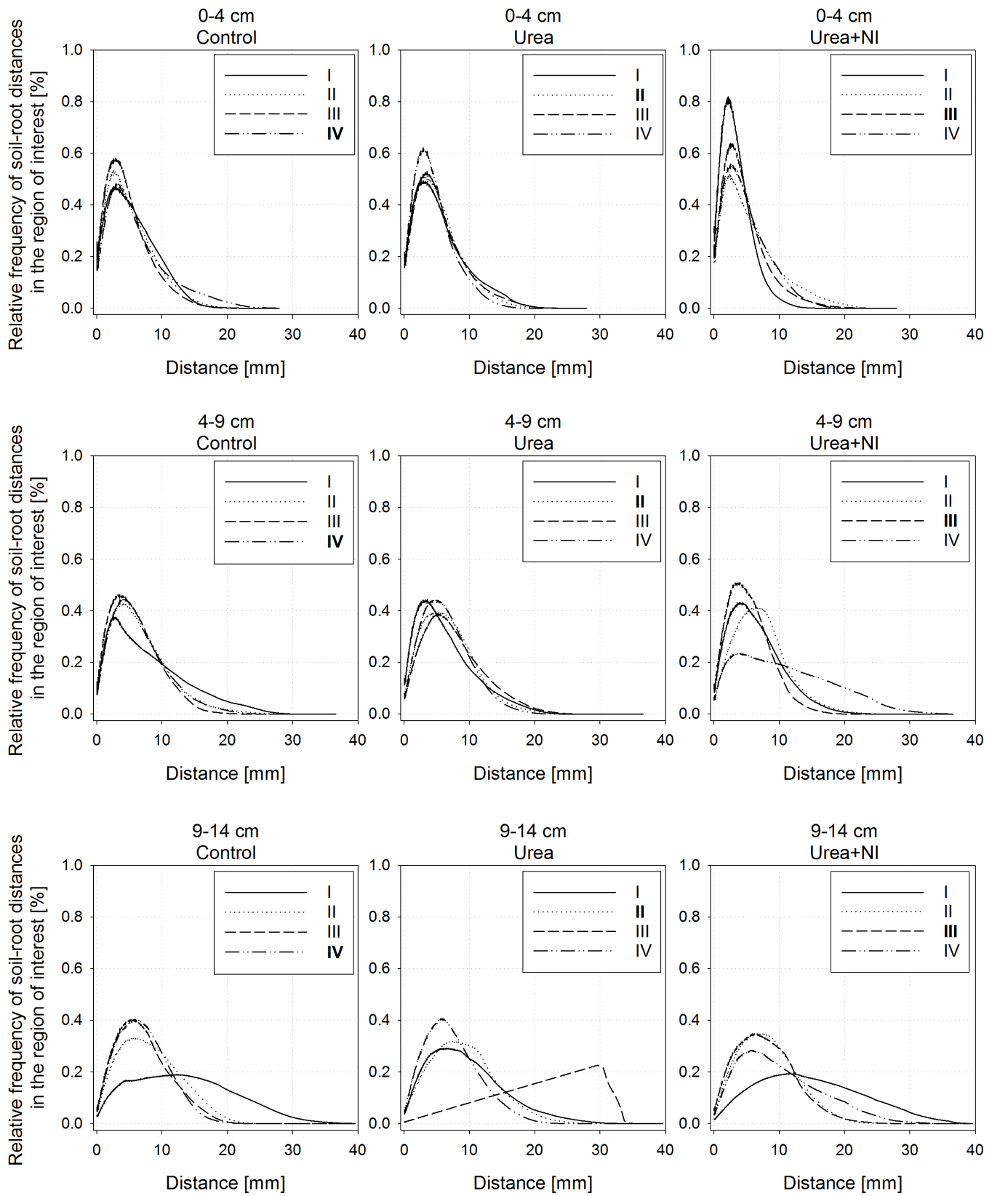


Figure S8: All relative frequencies [% of all soil voxels in the respective layer] of soil-root distances at 16 DAP for *Vicia faba*, separated in 3 layers, representing the fertiliser layer and both parts above and below the fertiliser layer. Replicate labelling refers to replicate numbering in Figure S6. Bold printed labels refer to the replicates closest to the treatment mean for root parameters selected for the presentation in Fig. 7.


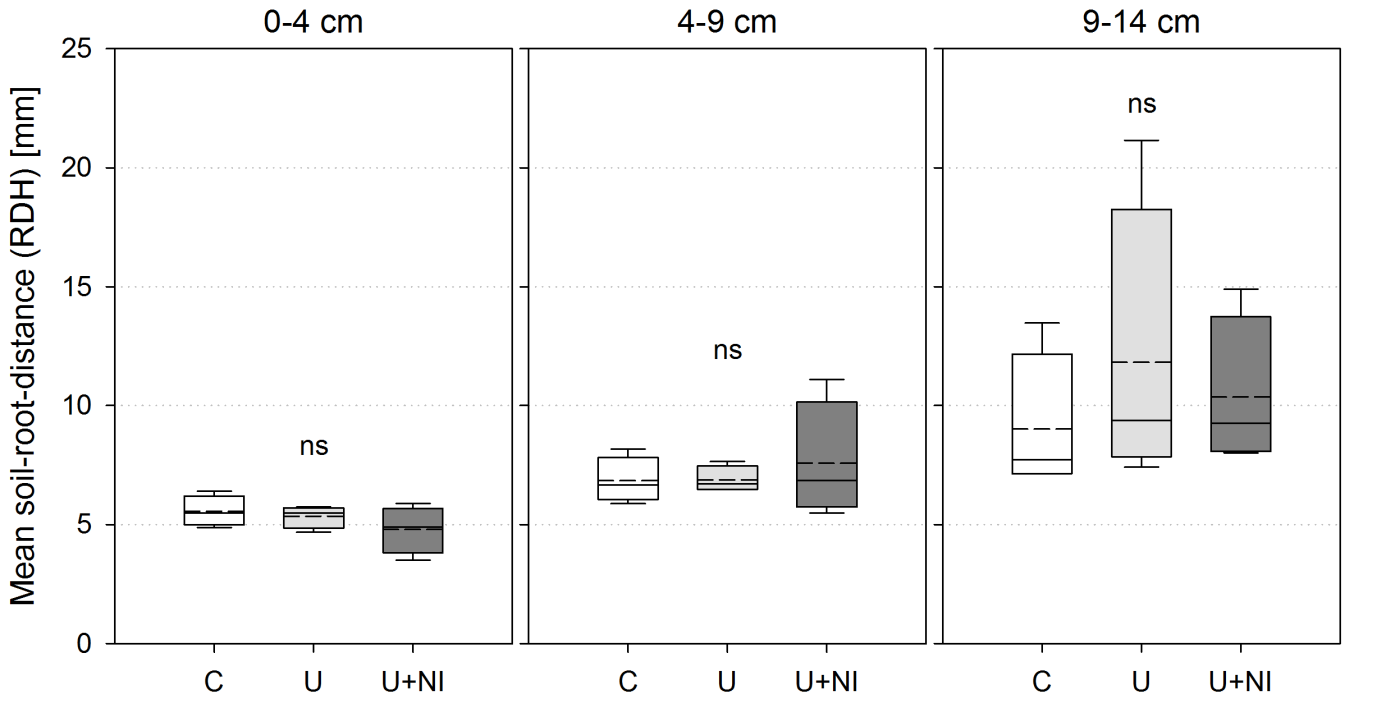


Figure S9: Mean soil-root-distances (RDH) at 16 DAP for *Vicia faba*, separated in 3 layers, representing the fertiliser layer and both parts above and below the fertiliser layer for the treatments control (“C”), urea (“U”) and urea with nitrification inhibitor (“U+NI”). Significant differences (*p*<0.05) between treatments are indicated by different letters, ns = not significant; dashed lines in the boxplots represent mean values while solid lines represent the median.


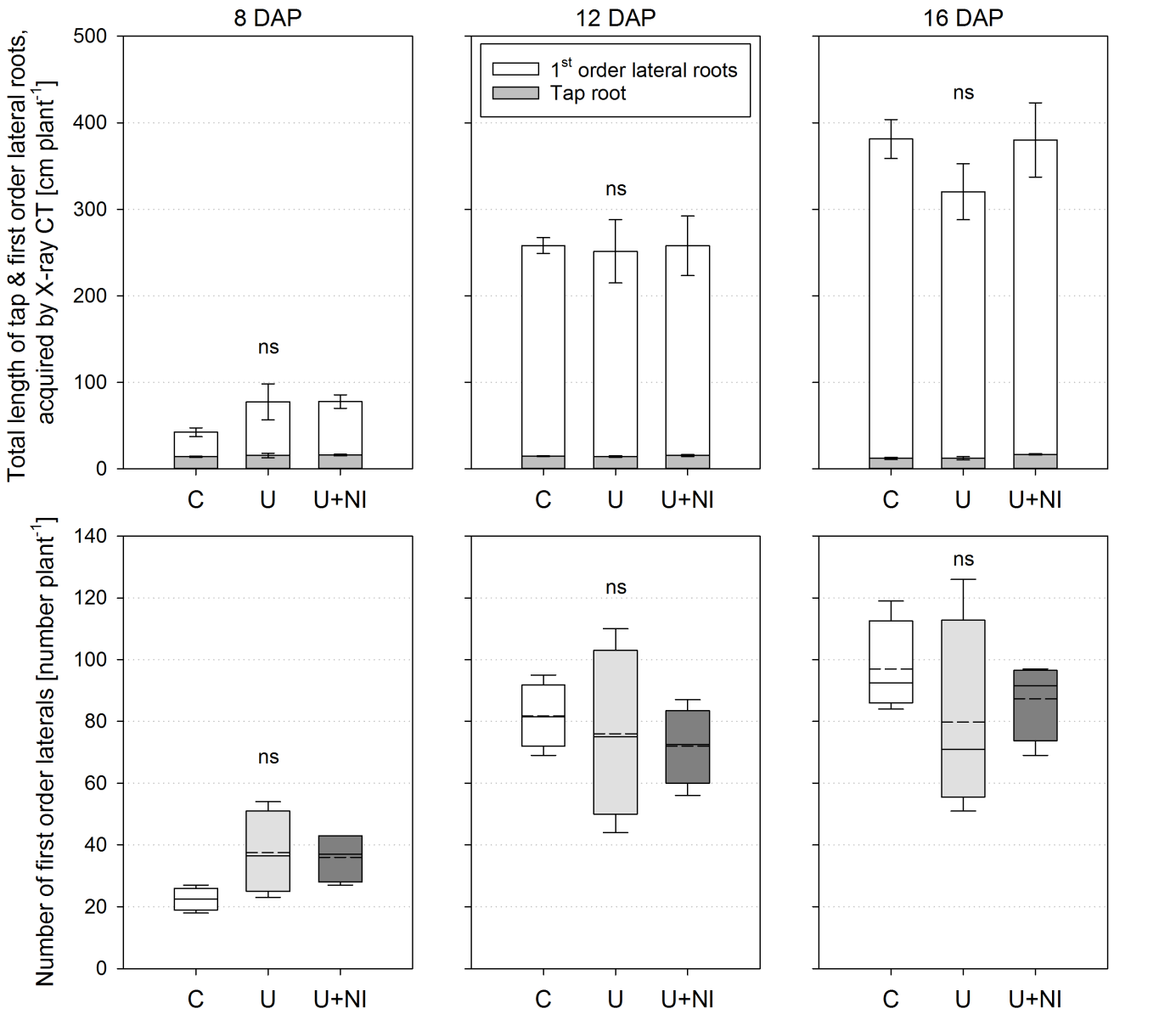


Figure S10: Root growth development of *Vicia faba* plants over time for the treatments control (“C”), urea (“U”) and urea with nitrification inhibitor (“U+NI”), acquired by X-ray CT. Upper row: white bars represent length of first order lateral roots per plant and grey bars represent length of tap root per plant. Bottom row: number of first order laterals per plant. Significant differences (*p*<0.05) between treatments are indicated by different letters, ns = not significant; dashed lines in the boxplots represent mean values while solid lines represent the median.


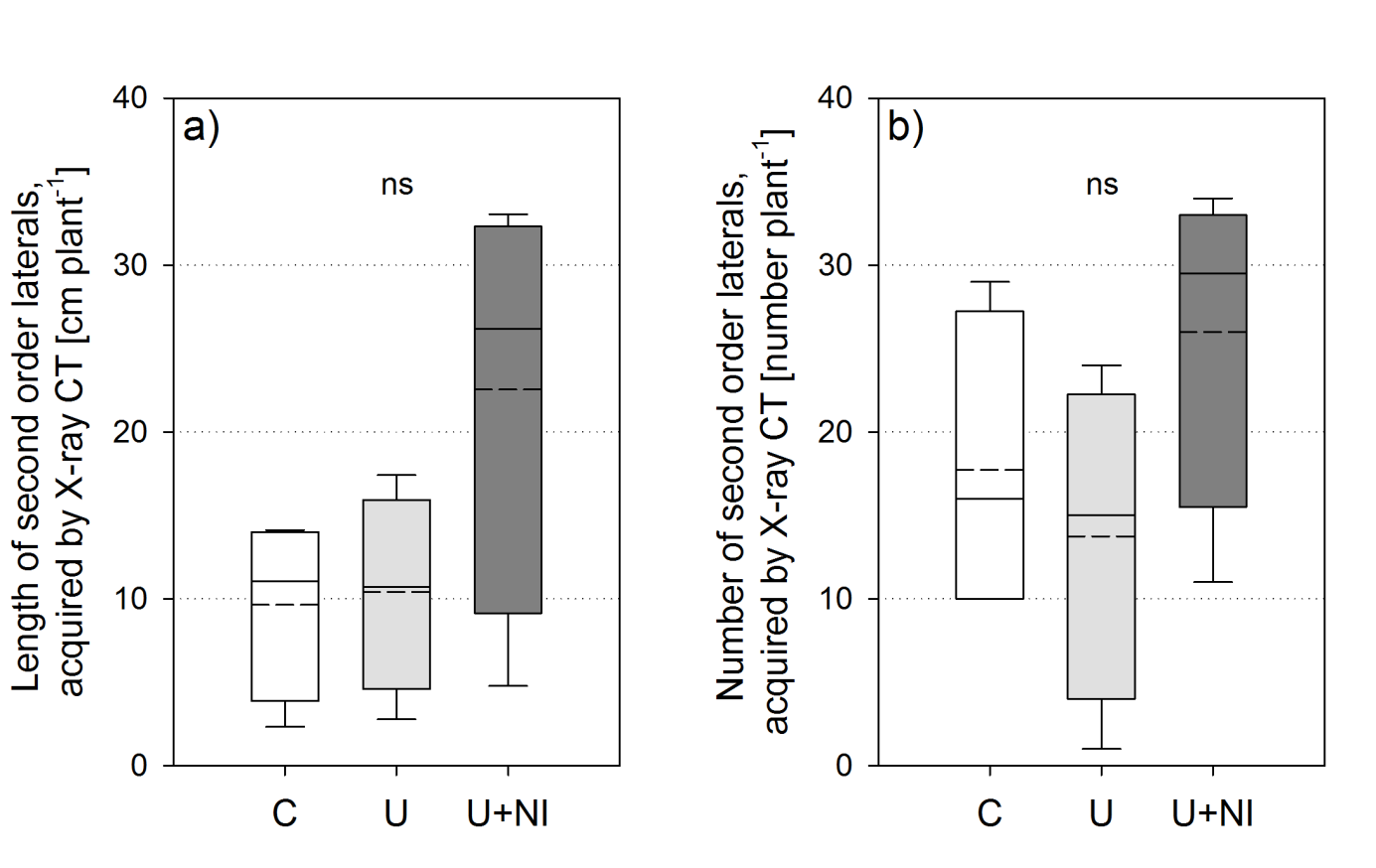


Figure S11: a) Length and b) number of second order lateral roots per plant of *Vicia faba* after 16 days of growth for the treatments control (“C”), urea (“U”) and urea with nitrification inhibitor (“U+NI”), acquired by X-ray CT. Significant differences (*p*<0.05) between treatments are indicated by different letters, ns = not significant; dashed lines in the boxplots represent mean values while solid lines represent the median.


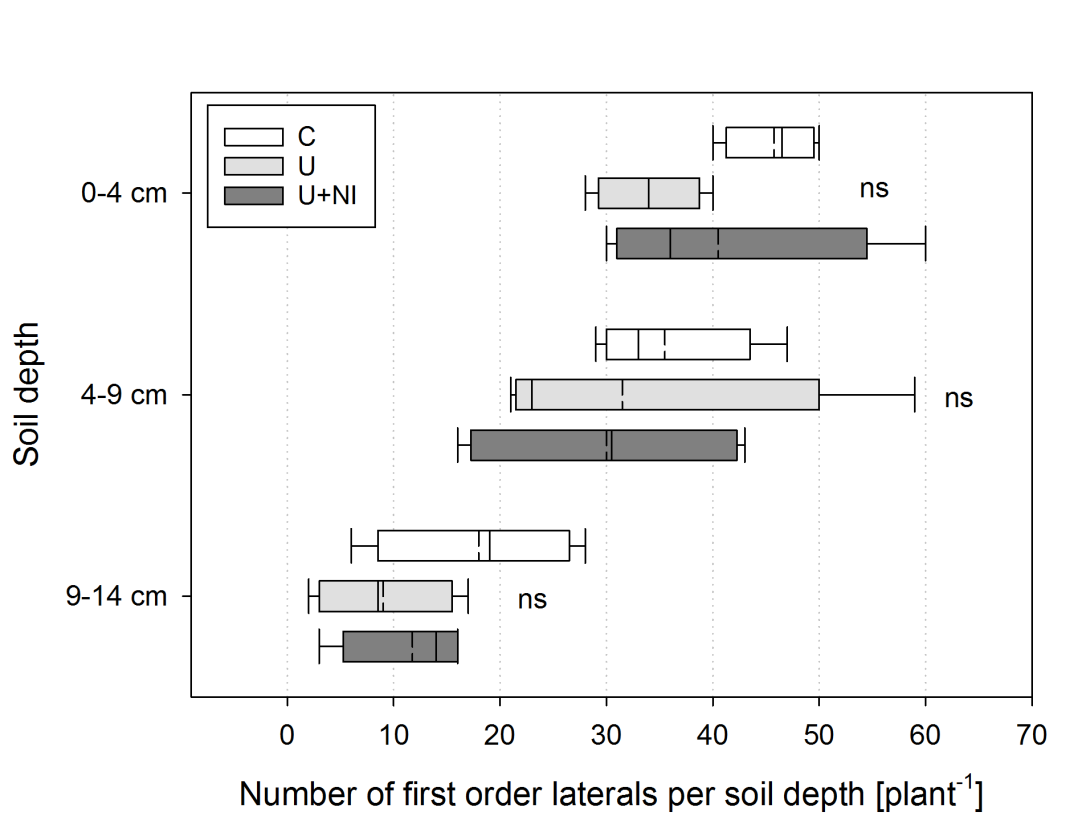


Figure S12: Depth distribution of number of first order laterals from *Vicia faba* after 16 days for the treatments control (“C”), urea (“U”) and urea with nitrification inhibitor (“U+NI”), acquired from X-ray CT data. Significant differences (*p*<0.05) between treatments are indicated by different letters, ns = not significant; dashed lines in the boxplots represent mean values while solid lines represent the median.


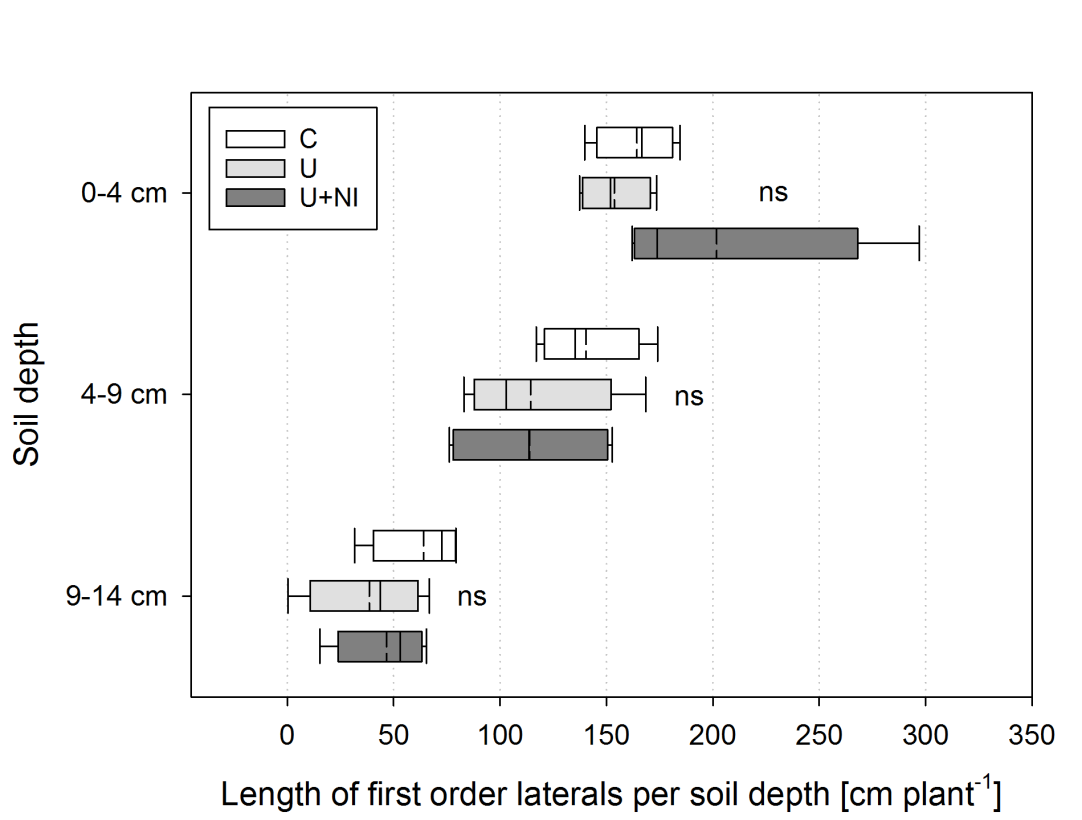


Figure S13: Depth distribution of first order lateral root length from *Vicia faba* after 16 days for the treatments control (“C”), urea (“U”) and urea with nitrification inhibitor (“U+NI”), acquired from X-ray CT data. Significant differences (*p*<0.05) between treatments are indicated by different letters, ns = not significant; dashed lines in the boxplots represent mean values while solid lines represent the median.


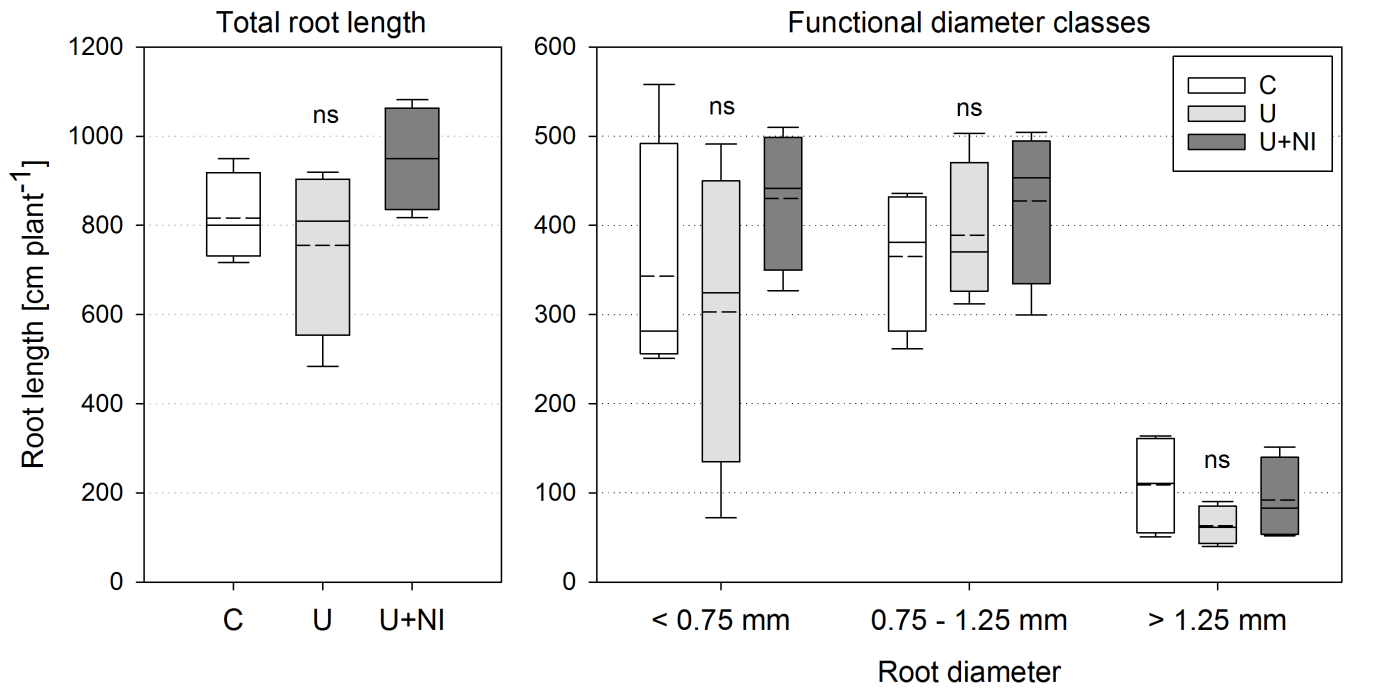


Figure S14: Total root length and root length per functional diameter classes (referring to root orders: second order laterals, first order laterals and tap root) for *Vicia faba* after 16 days for the treatments control (“C”), urea (“U”) and urea with nitrification inhibitor (“U+NI”), acquired by WinRHIZO. Significant differences (*p*<0.05) between treatments are indicated by different letters, ns = not significant; dashed lines in the boxplots represent mean values while solid lines represent the median.


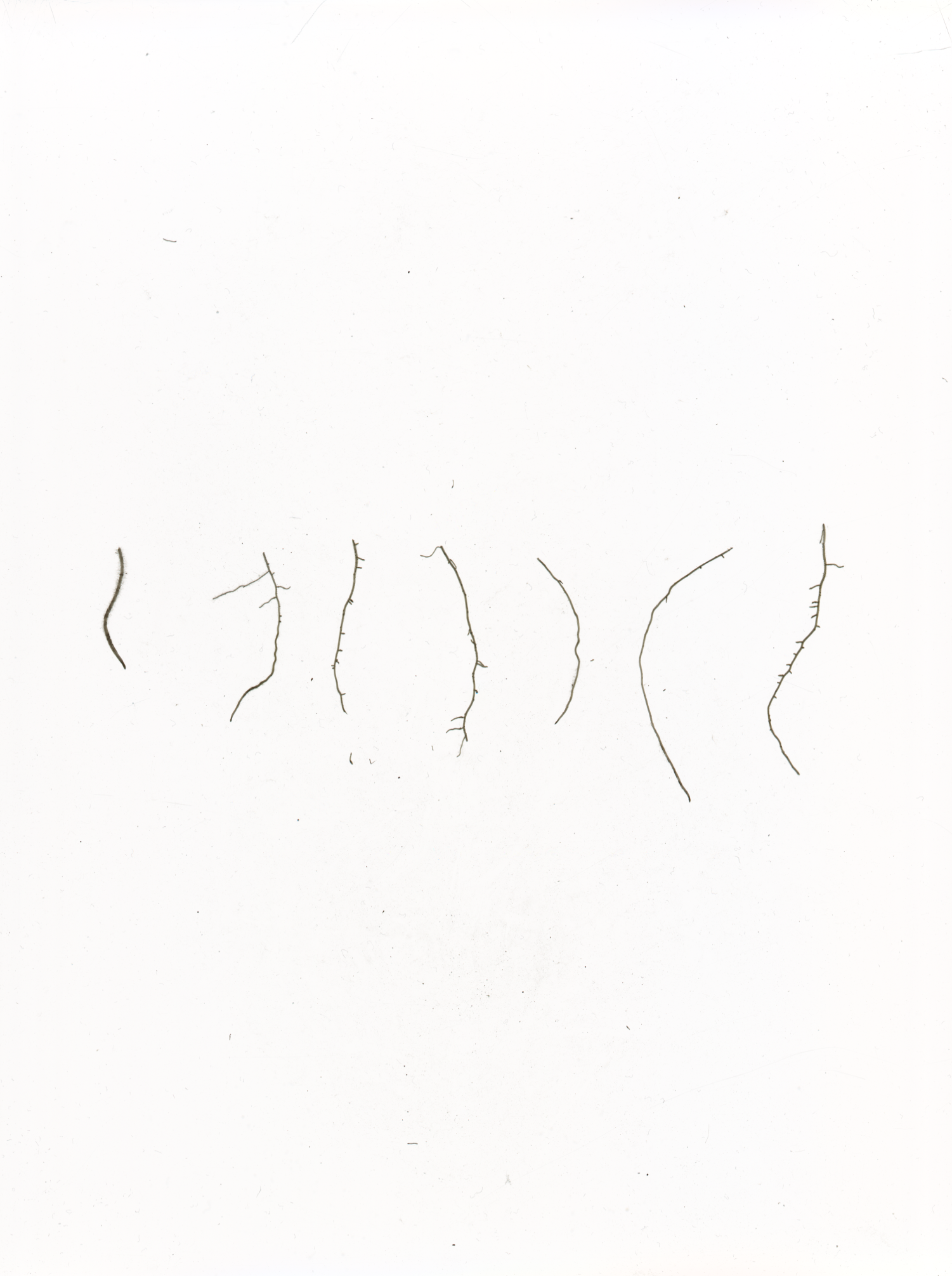


Figure S15: Example of root growth inhibition by high NO_3_^-^ in *Hordeum vulgare* (’Marthe’). Scanned roots in the fertiliser layer in the treatment U, 16 days after planting.
